# Dual-specificity phosphatase 3 deletion promotes obesity, non-alcoholic steatohepatitis and hepatocellular carcinoma

**DOI:** 10.1038/s41598-021-85089-6

**Published:** 2021-03-12

**Authors:** Sophie Jacques, Arash Arjomand, Hélène Perée, Patrick Collins, Alice Mayer, Arnaud Lavergne, Marie Wéry, Myriam Mni, Alexandre Hego, Virginie Thuillier, Guillaume Becker, Mohamed Ali Bahri, Alain Plenevaux, Emmanuel Di Valentin, Cécile Oury, Michel Moutschen, Philippe Delvenne, Nicolas Paquot, Souad Rahmouni

**Affiliations:** 1 Laboratory of Animal Genomics, GIGA-Medical Genomics, GIGA-Institute, University of Liège, B34, 1, Avenue de l’hôpital, 4000 Liège, Belgium; 2 Department of Pathology, Liège University Hospital, Liège, Belgium; 3 GIGA-Genomics Core Facility, GIGA-Institute, University of Liège, Liège, Belgium; 4 GIGA-Imaging Core Facility, GIGA-Institute, University of Liège, Liège, Belgium; 5 GIGA-CRC-In Vivo Imaging, GIGA-Institute, University of Liège, Liège, Belgium; 6 GIGA-Viral Vectors Core Facility, GIGA-Institute, University of Liège, Liège, Belgium; 7 Laboratory of Cardiology, GIGA-Cardiovascular Sciences, GIGA-Institute, University of Liège, Liège, Belgium; 8 Infectious Diseases Department, Liège University Hospital, Liège, Belgium; 9 Division of Diabetes, Nutrition and Metabolic Diseases, Department of Medicine, CHU Sart-Tilman and GIGA-I3, Immunometabolism and Nutrition Unit, University of Liège, Liège, Belgium

**Keywords:** Non-alcoholic fatty liver disease, Metabolic syndrome, Insulin signalling, Mechanisms of disease

## Abstract

Non-alcoholic fatty liver disease (NAFLD) is the most common chronic hepatic pathology in Western countries. It encompasses a spectrum of conditions ranging from simple steatosis to more severe and progressive non-alcoholic steatohepatitis (NASH) that can lead to hepatocellular carcinoma (HCC). Obesity and related metabolic syndrome are important risk factors for the development of NAFLD, NASH and HCC. DUSP3 is a small dual-specificity protein phosphatase with a poorly known physiological function. We investigated its role in metabolic syndrome manifestations and in HCC using a mouse knockout (KO) model. While aging, DUSP3-KO mice became obese, exhibited insulin resistance, NAFLD and associated liver damage. These phenotypes were exacerbated under high fat diet (HFD). In addition, DEN administration combined to HFD led to rapid HCC development in DUSP3-KO compared to wild type (WT) mice. DUSP3-KO mice had more serum triglycerides, cholesterol, AST and ALT compared to control WT mice under both regular chow diet (CD) and HFD. The level of fasting insulin was higher compared to WT mice, though, fasting glucose as well as glucose tolerance were normal. At the molecular level, HFD led to decreased expression of DUSP3 in WT mice. DUSP3 deletion was associated with increased and consistent phosphorylation of the insulin receptor (IR) and with higher activation of the downstream signaling pathway. In conclusion, our results support a new role for DUSP3 in obesity, insulin resistance, NAFLD and liver damage.

## Introduction

Non-alcoholic fatty liver disease (NAFLD) is strongly associated with obesity, insulin resistance, type 2 diabetes mellitus (T2DM), and cardiovascular complications and thus, is considered as the hepatic manifestation of metabolic syndrome. With the rapidly rising incidence of obesity and associated metabolic syndrome, NAFLD has become the most frequent chronic liver disease in Western countries, representing an important public health problem^1,2^. NAFLD includes a wide spectrum of liver clinicopathologic conditions, ranging from pure fatty liver or steatosis (fat infiltration in > 5% of hepatocytes) which is apparently a benign condition, to non-alcoholic steatohepatitis (NASH), which may progress to cirrhosis, liver failure, and hepatocellular carcinoma (HCC)^1^. The mechanisms involved in NAFLD progression include the development of steatosis, followed by increased inflammation, necrosis and activation of the fibrogenic cascade. This leads to NASH which is characterized by a robust inflammation in the hepatic tissue, as well as by hepatocyte ballooning. NASH can progress to more severe and irreversible stages of the disease including fibrosis and cirrhosis^1^. Unfortunately, at present, the only effective interventions for NASH consist of caloric restriction, exercise and weight loss, though these lifestyle approaches have proven difficult to adhere to in common clinical practice^1^, underscoring the urgent need for better understanding the molecular mechanisms involved in disease progression to implement efficient new targeted therapies. In this regard, several signaling molecules, including dual-specificity phosphatases (DUSPs), have been reported to regulate NAFLD development. Indeed, several studies have shown that various DUSPs are downregulated in the fatty liver^3–7^.

DUSP3 is a member of the DUSPs proteins family, which dephosphorylate the threonine/serine and tyrosine residues of their substrates^8^. Known substrates of DUSP3 include the MAPKs ERK and JNK, STAT5 and ErbB2^9^. It has also been reported to play a role in PKC signaling. DUSP3 activity is positively regulated by phosphorylation of Tyr138 by the tyrosine kinases ZAP-70 and Tyk2. Its activity can also be increased after association with the serine/threonine kinase VRK3^10^. DUSP3 plays an important role in the regulation of the cell cycle. This finding prompted investigation of its potential role in cancer. Indeed, DUSP3 expression is upregulated in cervix^11^ and prostate^12^ cancers as well as in several cancer-derived cell lines^11,12^ and is downregulated in breast^13^ and non-small cell lung cancers^14^. Using full DUSP3-KO mice, we uncovered unexpected roles of this phosphatase in endothelial cells and neo-angiogenesis^15^, in monocytes and macrophages^16,17^ and in platelets^18^. DUSP3-KO females, but not males, were shown to be resistant to multi-microbial and to lipopolysaccharides-induced septic shock. This protection was estrogen and M2-like macrophage dependent^16,17^. The role of DUSP3 in monocytes/macrophages function was further confirmed by the fact that DUSP3 KO favors Lewis lung carcinoma-induced macrophage recruitment at the tumor site, enhancing pulmonary metastases^19^. DUSP3 also plays a key role in arterial thrombosis through a mechanism involving GPVI and CLEC-2 signaling pathways, but seems to be dispensable for primary hemostasis^18^. It is noteworthy that these phenotypes were only observed following the application of specific challenges. Otherwise, DUSP3-KO mice are healthy at birth and do not spontaneously develop any pathology until 6 months of age. However, we observed that past this age, DUSP3-KO mice progressively become obese and develop NAFLD. This prompted us to further investigate the role of DUSP3 phosphatase in obesity, NAFLD and HCC.

## Results

### The genetic deletion of DUSP3 promotes obesity

**Figure 1:**
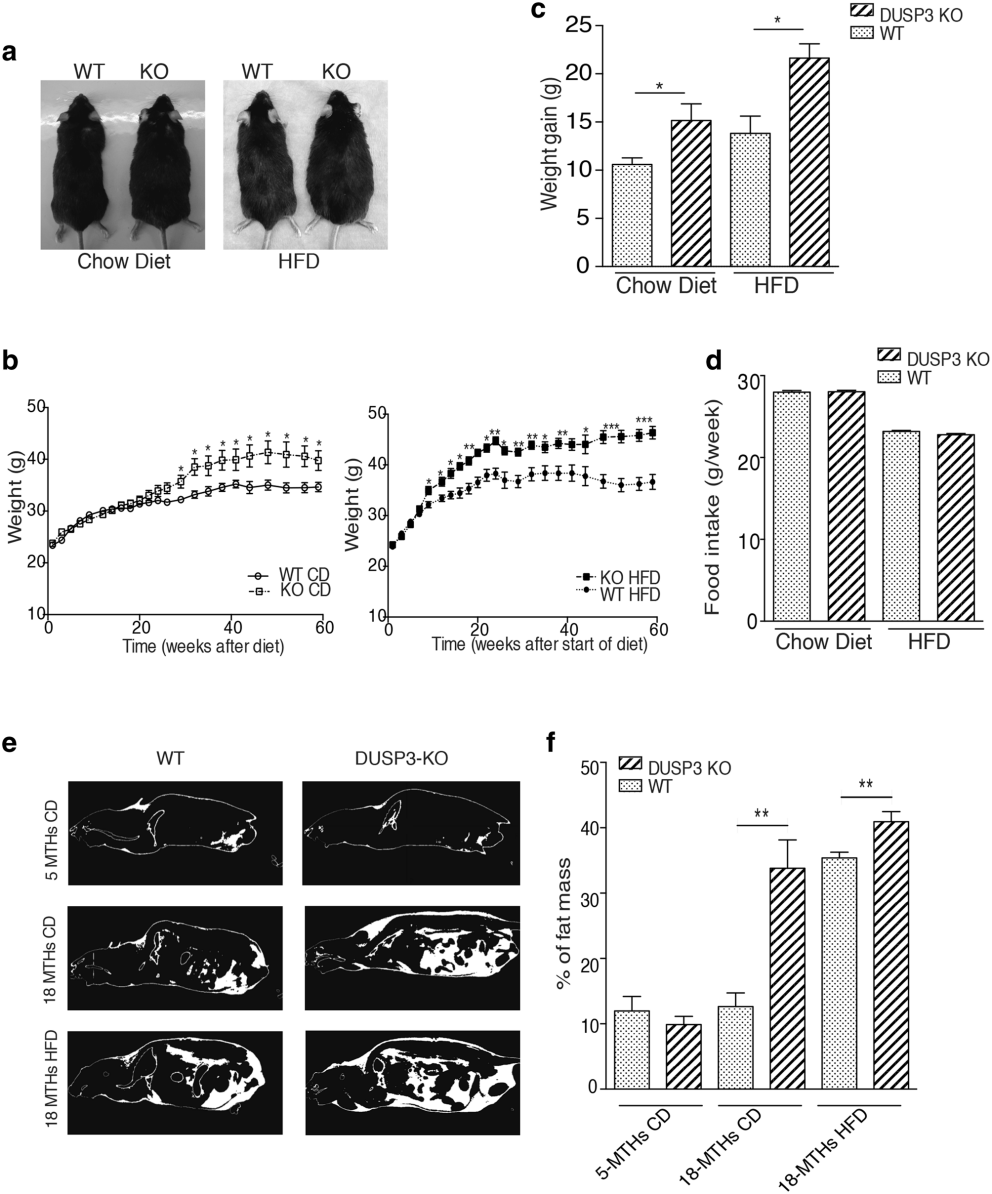
DUSP3 genetic deletion promotes obesity. (**a**) Representative images of 18-month-old WT and DUSP3-KO mice fed CD (left photo) and 5-month-old WT and DUSP3-KO mice fed HFD (right photo). (**b**) WT and KO mice were fed chow diet (left panel) or HFD (right panel) and body weight was monitored weekly during 60 weeks. The data are expressed as means ± SD (n = 10 mice in each group). (**c**) Weight gain of WT and KO mice after 60 weeks of CD or HFD. (**d**) Food intake during 27 weeks for WT and KO mice under CD and HFD. (**e**) Representative CT-scan images of 5- and 18-month-old WT and KO mice under CD and HFD. The white color represents fat and dark represent lean mass. (**f**) Quantification of fat body mass [n = 3 mice in each group shown in (**e**)] Data represent the mean ± SD of at least three mice of each group (*P < 0.5; **P < 0.01).

**Figure 2:**
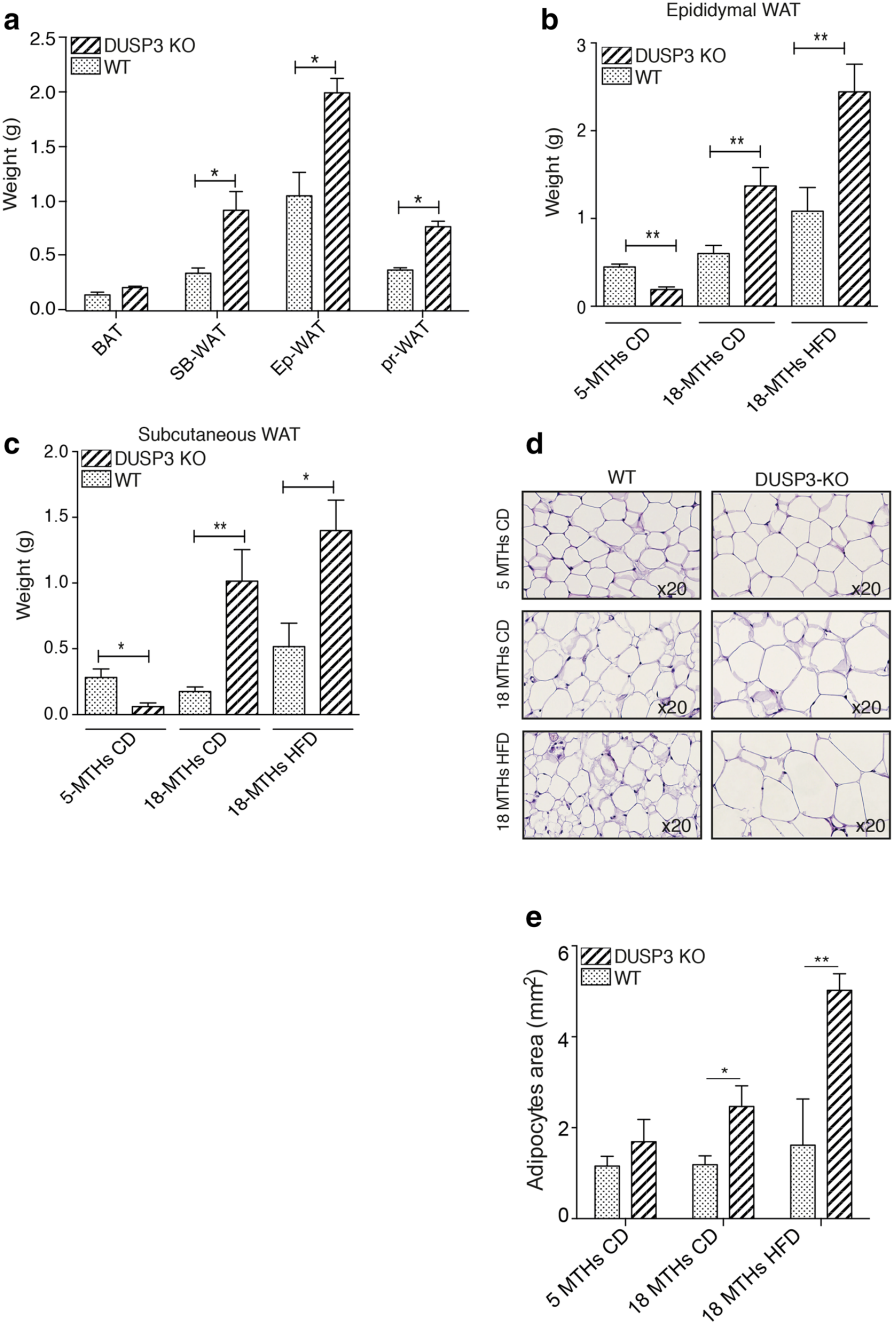
DUSP3 deletion promotes white adipose tissue accumulation. (**a**) White and brown adipose tissue (WAT and BAT, respectively) was assessed in 18-month-old WT and DUSP3-KO mice. *SB* subcutaneous, *Ep* epididymal and *Pr* perirenal. (**b**,**c**) Subcutaneous and epididymal fat assessment in 5-month-old WT and KO mice under CD and in 18-month-old WT and KO mice under CD and HFD. (**d**) Representative paraffin sections of epididymal WAT from 5-month-old WT and DUSP3-KO mice under CD and at 18-month-old under CD and HFD. Sections were stained with H&E. Magnification: ×20. (**e**) Average adipocytes cell area of epididymal WAT of the mice shown in (**d**). Data represent the mean ± SD of at least three mice of each group (*P < 0.5; **P < 0.01).

We previously generated full, constitutive DUSP3 knockout mice (KO)^15^. While young (< 6 months old), these mice did not spontaneously develop any phenotype. However, when aging, the body weight of KO mice progressively exceeded that of WT mice of the same age fed regular chow diet (CD). This phenotype was enhanced under high fat diet (HFD) (Fig. 1a–c). Indeed, between 2 and 18 months of age, mutant mice gained 63% and 86% of their basal weight, while WT littermates gained 45% and 58% under CD and HFD respectively (Fig. 1c). The excess body weight of DUSP3-KO mice was not due to hyperphagia. Indeed, there was no significant difference in food intake between WT and KO groups as measured during a period of 27 weeks (from 4 to 10 months old) for both CD and HFD (Fig. 1d). The increased body weight of DUSP3 KO was at least partially due to a significant increase in adipose tissue. At 18 months of age, under CD, mutant mice had 153% more fat tissue than controls, whereas under HFD, mutant mice had 23% more (P_0.01, Fig. 1e,f). The weight of all white adipose tissues (WAT) was increased in DUSP3-KO compared to WT under both CD and HFD, but not that of brown adipose tissue (BAT) (Fig. 2a–c). The size of adipocytes was significantly increased in DUSP3-KO mice under both CD and HFD compared to WT mice (Fig. 2d,e).

### DUSP3 deletion promotes NAFLD, NASH, fibrosis and HCC under HFD

**Figure 3:**
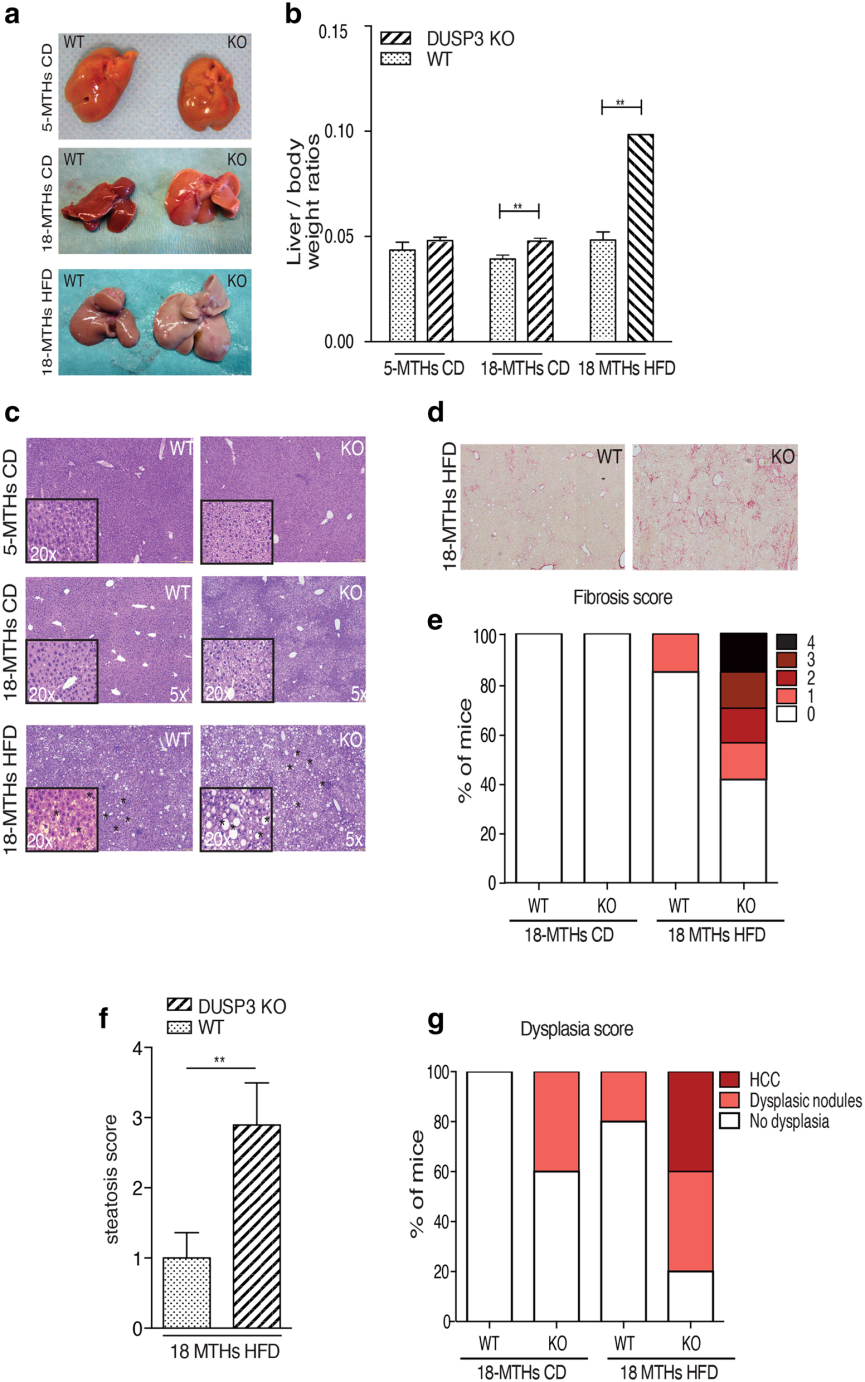
DUSP3 deletion promotes NAFLD and associated liver damages. (**a**) Representative image of liver from mutant and WT mice fed CD or HFD. (**b**) Quantification of liver to body weight ratio. Data represent the mean ± SD of at least ten mice of each of the indicated group (**P < 0.01). (**c**) Representative images of H&E staining of liver sections from DUSP3-KO and WT fed CD or HFD. Asterisks indicate representative lipid droplets. (**d**) Representative images of Sirius Red staining of liver sections from DUSP3-KO and WT fed HFD. (**e–g**) Fibrosis (**e**), steatosis (**f**) and dysplasia (**g**) scores. Mean ± SD values for all mice are shown. *P < 0.05; **P < 0.01.

Obesity is often accompanied by the development of NAFLD. Indeed, in addition to the significant increase of adipose mass tissue in DUSP3-KO mice, the ratio of liver to body weight was also significantly increased compared to WT mice under both CD and HFD (Fig. 3a,b). In KO mice, the liver also became lighter in color (Fig. 3a). Staining liver sections with H&E revealed the accumulation of lipid droplets in hepatocytes of HFD-fed mice (Fig. 3c). In addition, DUSP3 KO combined with HFD increased liver damage, with the appearance of fibrosis, dysplasia and even HCC. Indeed, 66% of mutant mice developed fibrosis (2 mice with lowest score (1) and the rest (1 mouse each) with score 2, 3 and 4) while only 16% of WT mice did (1 mouse out of 6 with the lowest score (1) (Fig. 3d,e). Steatosis score was similarly higher in KO  compared to WT mice (Fig. 3f). Dysplastic nodules and HCC developed in 40% of the mutant mice but in none of WT mice (Fig. 3g). Consistently, expression level of pro-fibrotic genes such as *Col1a1* (coding for collagen II) *Timp1* (coding for Timp1) were increased in DUSP3-KO mice under HFD compared to WT mice (data not shown).

**Figure 4:**
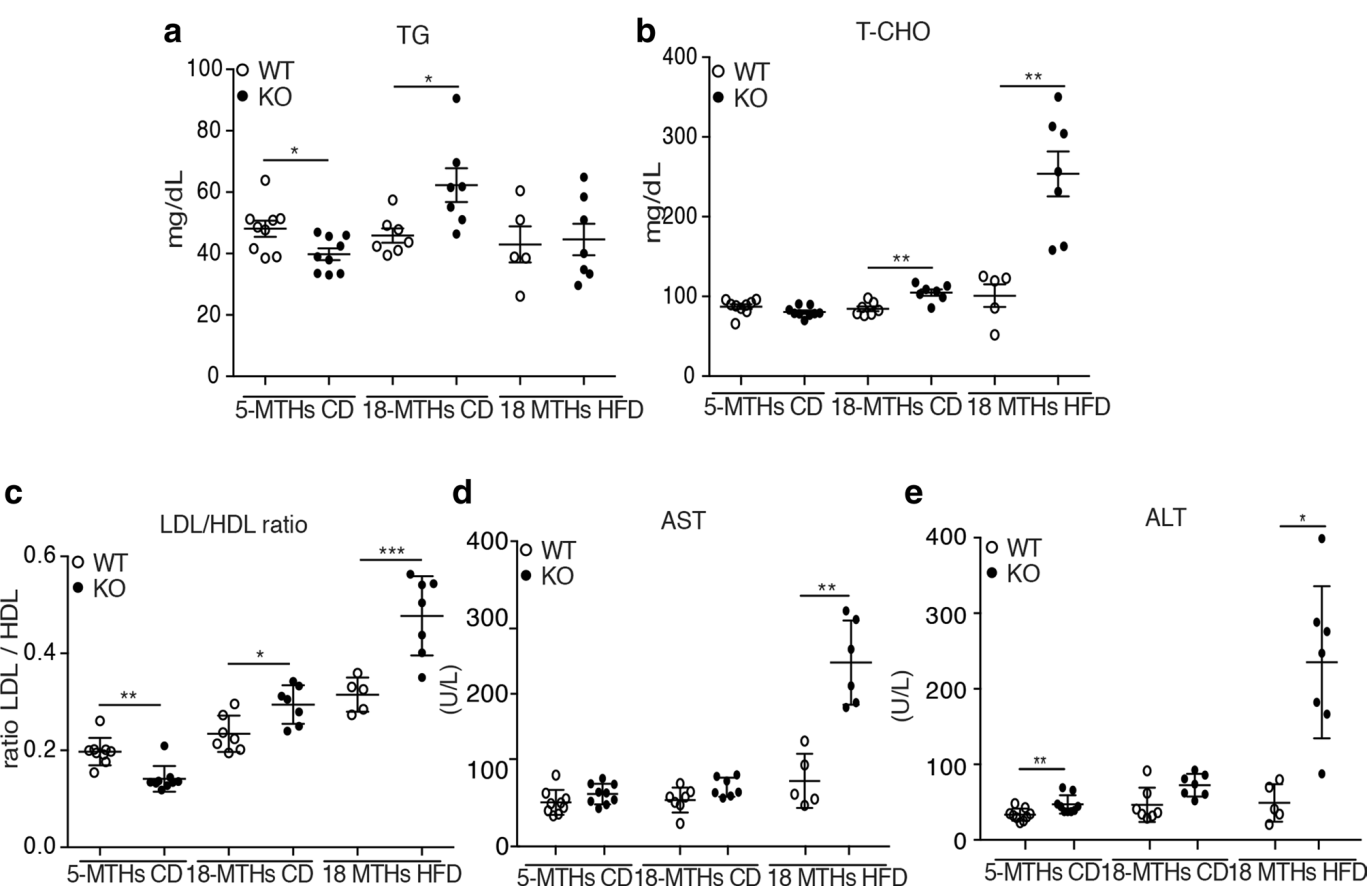
DUSP3 deletion promotes NAFLD and associated liver damages. Concentrations of TG (**a**), T-CHO (**b**), LDL/HDL ratio (**c**), AST (**d**) and ALT (**e**) in the sera of mice. Each dot represents one mouse. Mean ± SD values for all mice are shown. *P < 0.05; **P < 0.01; ***P < 0.001.

Consistently, DUSP3-KO exhibited higher levels of circulating triglycerides (TG) at the age of 18 months under HFD as well as serum total cholesterol (T-CHO). Levels of HDL decreased while no difference was observed for the LDL level at the same age. Consequently, the ratio of LDL on HDL was increased in KO mice compared to WT mice under both diets (Fig. 4a–c and Supplementary Fig. S1a,b). Alanine transaminase (ALT) and aspartate transaminase (AST), considered as important markers of NAFLD, were also increased in mutant mice compared to WT mice under CD and HFD (Fig. 4d,e). Interestingly, young mice (5 months under CD) did not exhibit such phenotypes. Indeed, we did not observe any liver associated damage. On the contrary, young mutant mice were even leaner than their WT littermates, they had no steatosis and TG levels as well as LDL/HDL ratio were significantly decreased in these mice (Fig. 4a–c). These results demonstrate that deletion of DUSP3 triggered lipid accumulation in aging mice.

### DUSP3 deletion associated with HFD strongly promote DEN-Induced hepatocarcinogenesis

**Figure 5:**
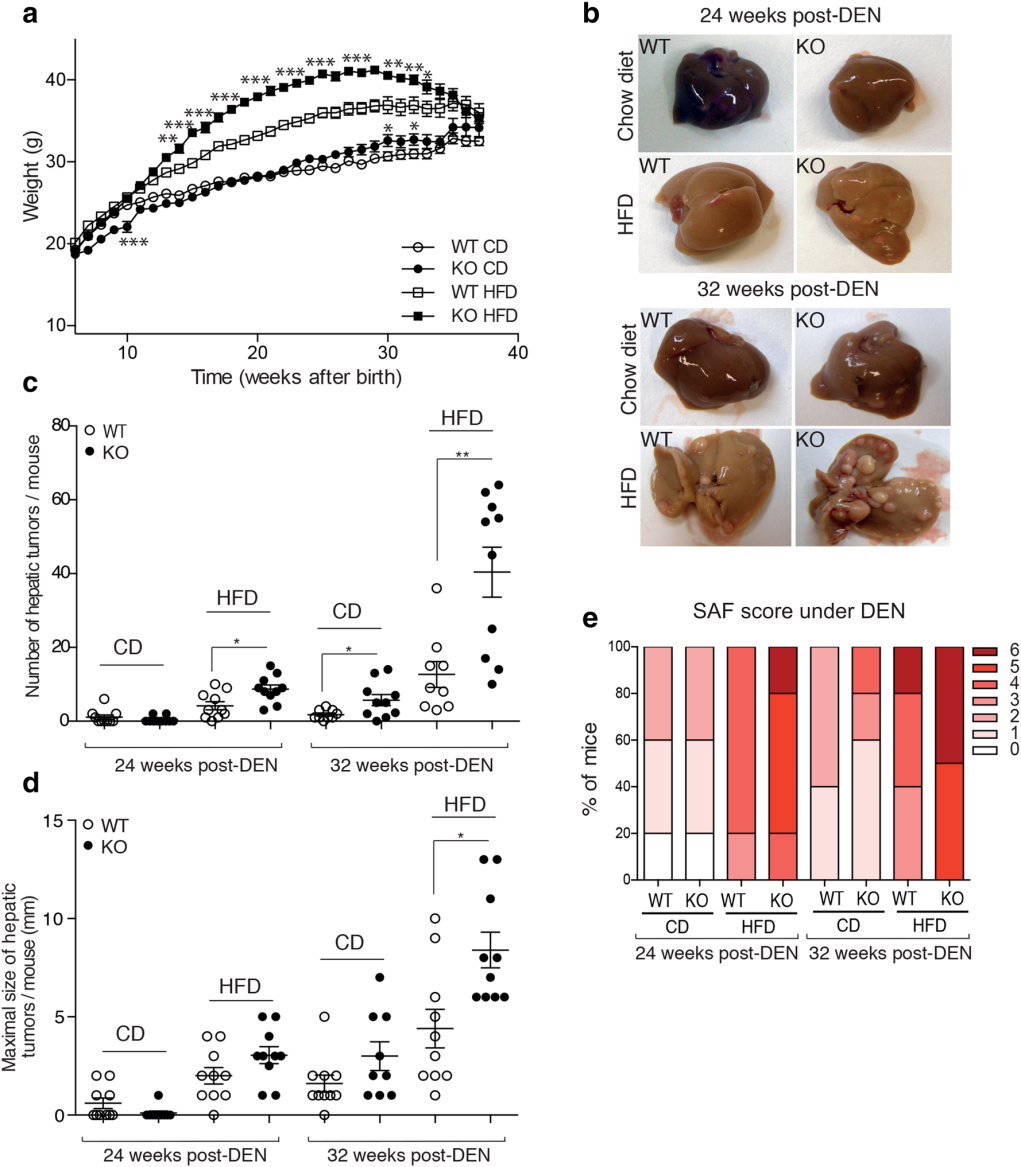
DUSP3 deletion associated with HFD strongly promote DEN-induced hepatocarcinogenesis. DEN (25 mg/kg) was i.p injected to 14-day-old DUSP3-KO and WT mice. Four weeks later, diet of the mice was switched to CD or HFD and weight was monitored every two weeks for 31 weeks (**a**). (**b**) Livers of WT and mutant mice kept on CD or HFD 24 and 32 weeks after the administration of DEN. (**c**–**e**) Tumor multiplicity (**c**), size (**d**), and SAF score evaluation (**e**) in livers of DEN-injected WT and DUSP3-KO mice kept on CD or HFD as above. Each dot represents the average number of tumors or size of the tumors for one mouse. Results are means ± SD. Each dot represents one mouse. Mean ± SD values for all mice are shown. *P < 0.05; **P < 0.01. n = 10 mice in each group.

Obesity and NAFLD are well-established risk factors for HCC^1^, although the corresponding tumor-promoting mechanisms are not well known. Given the observed spontaneous liver damage and hepatosteatosis in aged DUSP3-KO mice, we hypothesized that administration of the hepatic carcinogen, diethylnitrosamine (DEN)^20^ may induce HCC in DUSP3-KO mice. We placed DEN-injected WT and KO mice under either CD or HFD. Mice weight was monitored every week. We sacrificed the mice at 24 weeks and 32 weeks post-DEN injection. As expected, DUSP3-KO mice had significantly increased body weight compared to WT mice of the same age under CD and HFD (Fig. 5a). However, contrary to mice that did not receive DEN (Fig. 1a,b), the weight of mutant mice under DEN and HFD started to decline at 26 weeks post-DEN injection (Fig. 5a). Importantly, when analyzed at 26 and 32 weeks of age, DUSP3-KO mice kept on HFD exhibited many more and larger tumors per liver than WT mice (Fig. 5b–e and Supplementary Fig. S2a,b). We next used SAF score, the unweighted summation of semi-quantitative evaluation of steatosis, lobular inflammation and ballooning to which we added the evaluation of fibrosis, the appearance of dysplastic nodules and loss of liver architecture was also elevated in DUSP3-KO mice compared to WT littermates (Supplementary Fig. S1c). Mutant mice under DEN and CD or HFD showed higher SAF score compared to WT mice (Supplementary Fig. S2c). HFD and deletion of DUSP3 also increased serum TG, T-CHO and LDL/HDL ratio (Supplementary Fig. S2c–f), AST and ALT (Supplementary Fig. S3a,b).

### DUSP3 deficiency exacerbates HFD-induced insulin resistance

**Figure 6:**
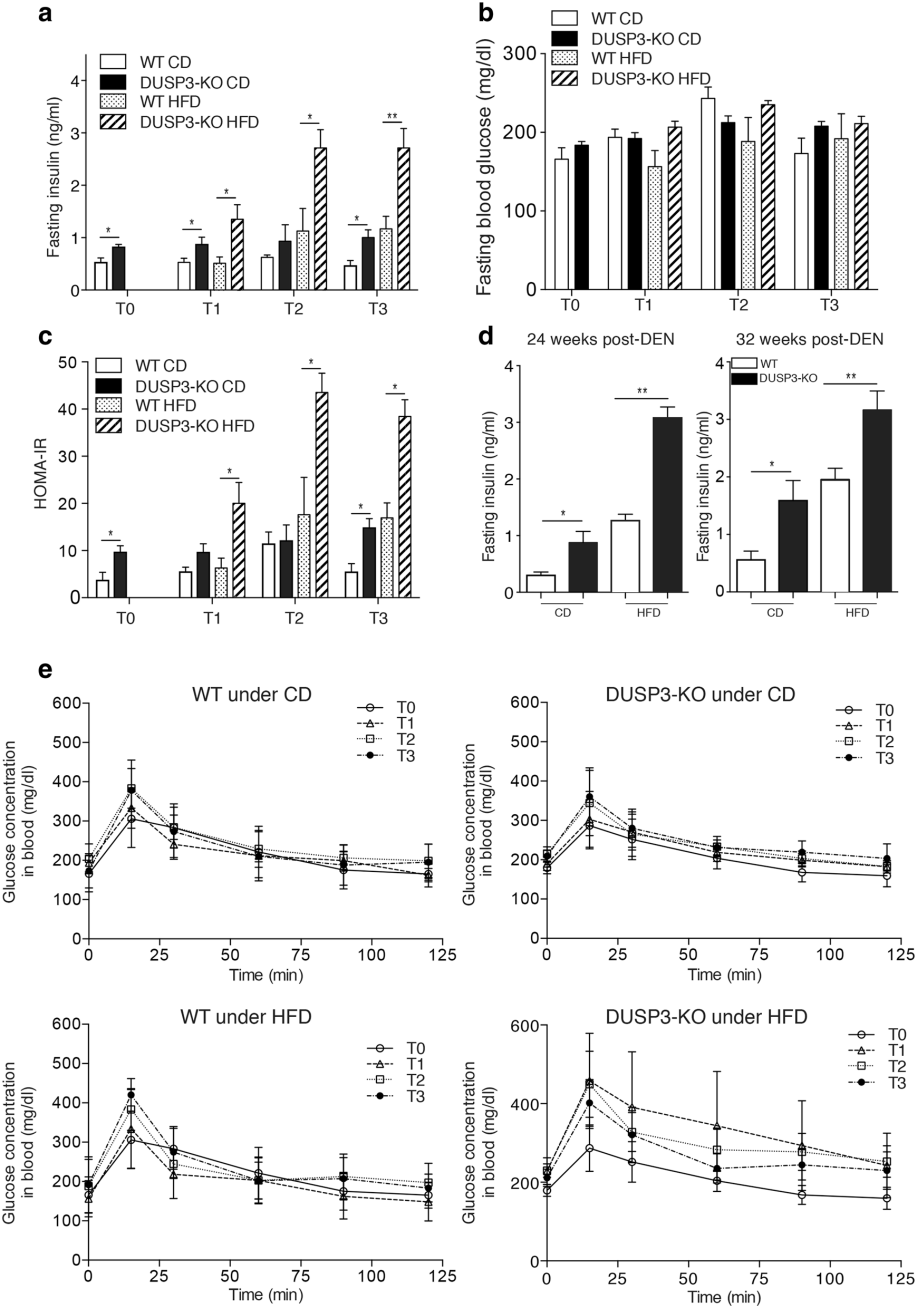
DUSP3 deletion exacerbate insulin resistance under HFD. Fasting blood insulin (**a**), blood glucose (**b**) and HOMA-IR (**c**) in DUSP3-KO and WT mice under CD or HFD. At all-time points, mice were starved for 6 h and blood was collected from tail vein to measure insulin and glucose. T0, basal level, corresponds to the measurement at the starting day of HFD. T1, T2 and T3 correspond respectively to 4 weeks, 12 weeks and 20 weeks under HFD or CD. N = 5 mice in each group of T1 to T3. N = 10 mice in each group at T0. (**d**) Fasting blood insulin in 24 weeks (left panel) and 32 weeks (right panel) after DEN challenge of DUSP3-KO and WT mice under CD or HFD. N = 5 mice in each group. Data represent means ± SD. Significance was determined by one-way ANOVA.

Insulin resistance is one of the pathognomonic features of obesity and NAFLD. We therefore investigated whether DUSP3 KO could exacerbate HFD-induced insulin resistance. We found that, even under CD, DUSP3 deletion was associated with a significant increase of plasma insulin levels at all time points analyzed, namely at 14, 18, 26, 34 weeks old without DEN (Fig. 6a) or at 24 and 32 weeks post-DEN injection (Fig. 6d). However, the effect of the DUSP3 deletion was more pronounced under HFD (Fig. 6a). Surprisingly, despite the high insulin levels, we did not observe any difference in fasting glucose levels between the different groups of mice under both CD and HFD (Fig. 6b). Though, the HOMA-IR, which assesses insulin resistance from fasting glucose and insulin levels, was significantly higher in DUSP3-KO compared to WT mice under both CD and HFD and all time points analyzed (Fig. 6c). Despite the observed insulin resistance in the KO mice, under CD, glucose homeostasis was not altered by DUSP3 deletion as assessed by the OGTT assay (Fig. 6e, top panels). Under HFD (Fig. 6e, lower panels), although not statistically significant, glucose level was higher and its clearance was slower over time in mutant compared to WT mice.

### DUSP3 expression is reduced under HFD and its genetic deletion enhances IR phosphorylation and signaling

**Figure 7:**
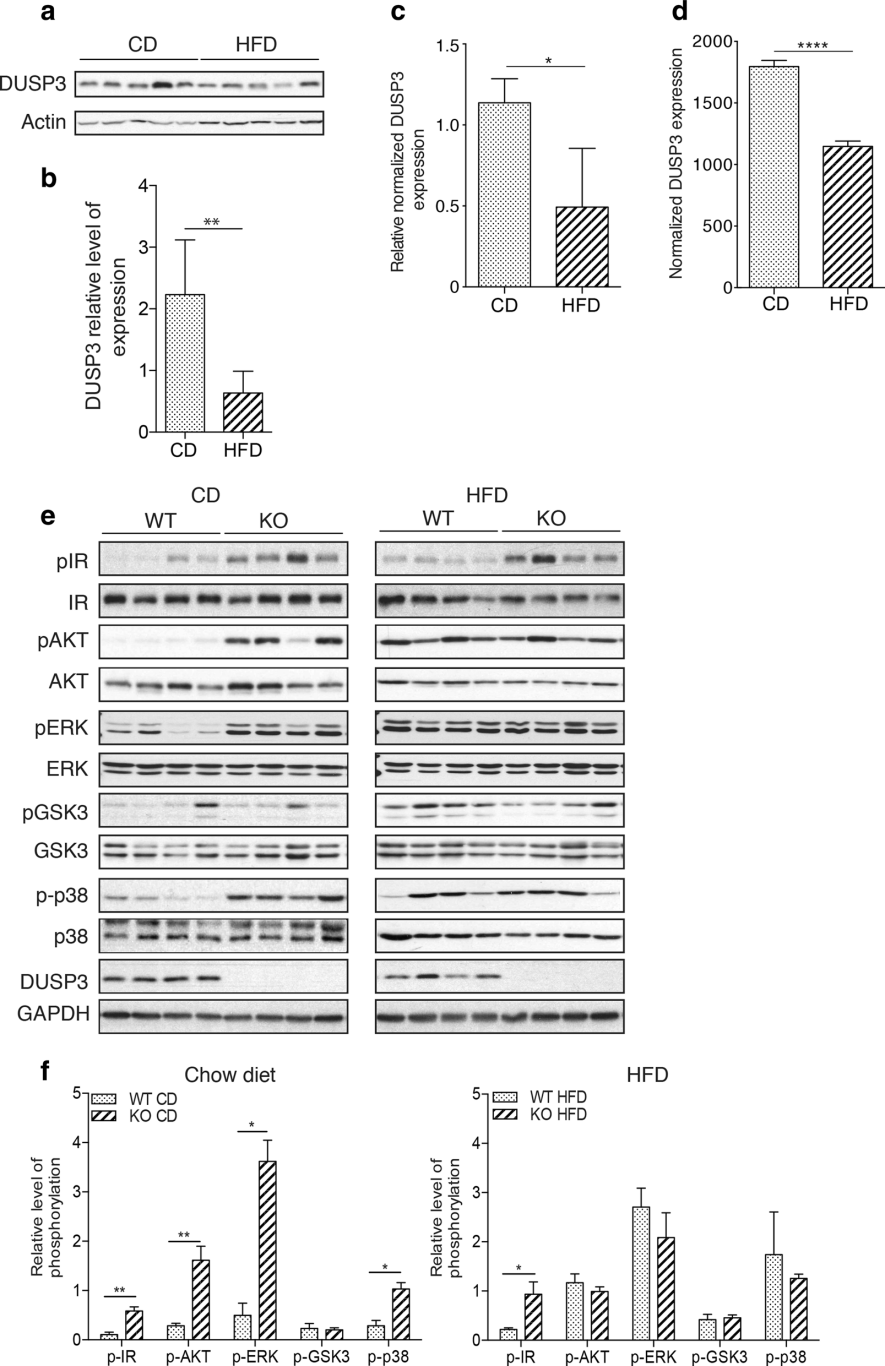
DUSP3 is involved in IR phosphorylation and signaling and its expression is reduced under HFD. (**a**–**d**) DUSP3 expression is reduced in mice liver under HFD. (**a**–**b**) DUSP3 protein expression. mRNA levels using qRT-PCR (**c**) and RNAseq (**d**) in liver extracts from 18-month-old WT mice under chow diet (CD) and high fat diet (HFD). (**a**) representative Western blot in liver samples of WT mice under CD and HFD. (**b**) Quantification of protein expression was normalized on beta-actin while (**c**) quantification of transcripts was normalized on HPRT housekeeping gene. Data are presented as mean ± SD. N = 3 mice in each group. (**e**–**f**) Protein level and phosphorylation of IR, AKT, ERK, GSK3 and p38 in the mouse liver tissue from DUSP3-KO and WT mice under CD and HFD. Liver homogenates protein extract were subjected to Western blot using anti-phospho IR, anti-phospho AKT, anti-phospho-ERK, anti-phospho GSK3 and anti-phospho p38. Anti-IR, AKT, ERK, GSK3 and p38 were used for normalization of each phosphorylation level. Global normalization was achieved using anti-b-actin antibody. (**e**) Representative blots are shown. (**f**) The average relative grayscale values normalized with the control protein were obtained from 4 to 6 mice per group and results are expressed as mean ± SD. *P < 0.05; **P < 0.01. Non-cropped original blots for each shown phospho-protein and protein are in the extended Supplementary Fig. 7 in the Supplementary Information.

**Figure 8:**
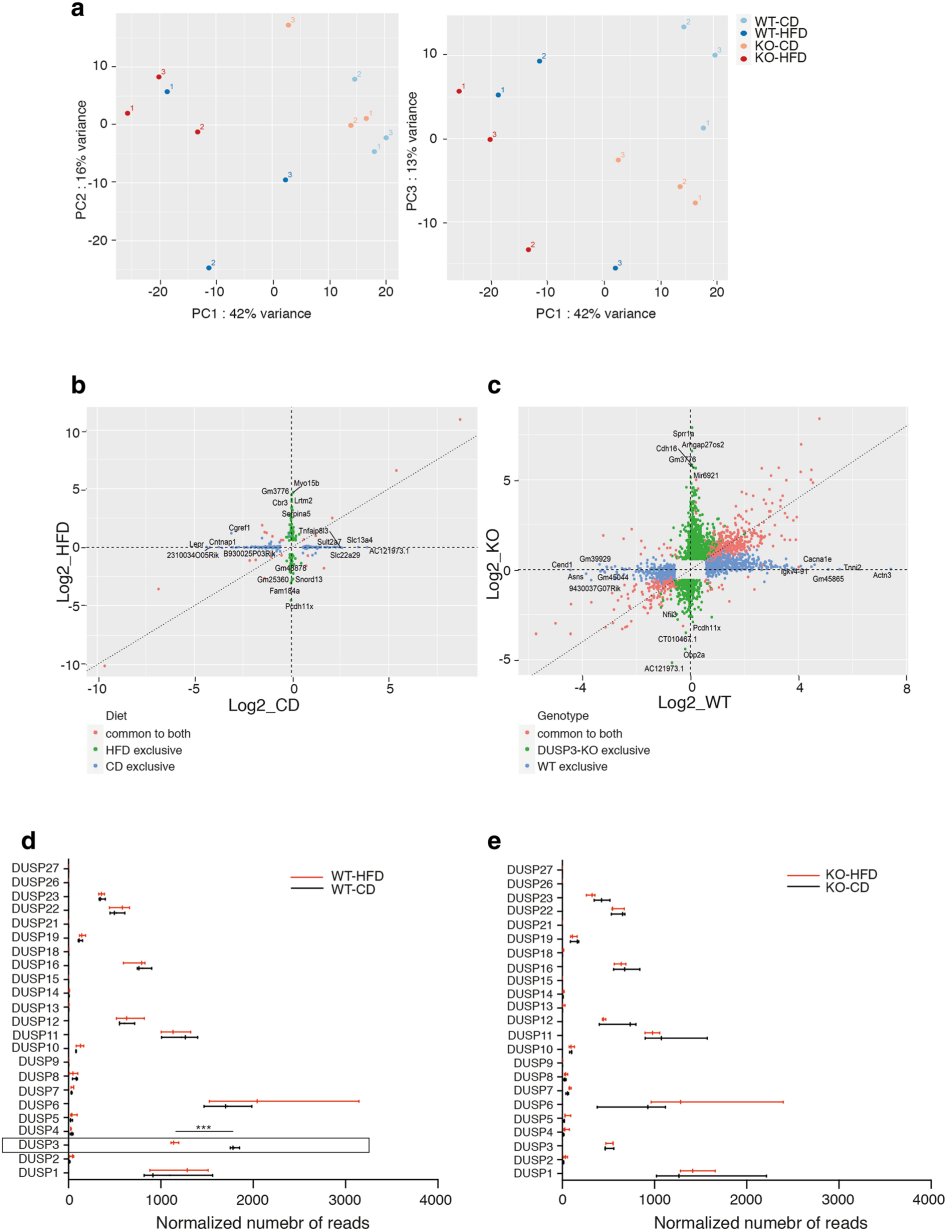
RNAseq analysis of DUSP3-KO and WT mice livers under CD and HFD. (**a**) Principal component analysis (PCA) on DUSP3-KO mice livers under CD (orange dots), or HFD (red dots) and WT mice livers under CD (light blue dots) or HFD (dark blue dots). (**b**,**c**) Comparison of the fold change difference between WT and DUSP3-KO genotypes for CD and HFD (**b**) and between CD and HFD for WT and the DUSP3-KO (**c**). Genes with adjusted p-value < 0.05 and fold change > 1.5 in at least one genotype were plotted. (**b**) Genes exclusive to CD are blue, the ones exclusive to HFD are green and the ones in common to both are red. (**c**) Genes exclusive to WT are blue, the ones exclusive to DUSP3-KO are green and the ones in common to both are red. Genes with the 5 highest or lowest fold changes in each group are labeled. (**d**,**e**) Comparison of expression levels of all known DUSPs in WT and DUSP3-KO (KO) mice under CD and HFD. Data are shown as normalized number of reads for each individual gene. ***P < 0.001.

To investigate DUSP3 function in NAFLD, we first evaluated its expression in liver samples from 18-month-old WT mice under CD or HFD. Expression of DUSP3 was significantly decreased in the livers of HFD fed mice (Fig. 7a,b). DUSP3 mRNA level was also significantly reduced under HFD as demonstrate by the qRT-PCR (Fig. 7c) and RNA-seq data (Figs. 7d, 8c and Supplementary Fig. S4). The insulin resistance observed in DUSP3-KO mice suggests that this phosphatase could play a role in insulin receptor (IR) signaling. We therefore investigated this pathway and observed that the IR was significantly hyper-phosphorylated in the absence of DUSP3 regardless of the type of diet, though, this hyper-phosphorylation was exacerbated under HFD (Fig. 7e,f). This hyper-phosphorylation of IR was accompanied by phosphorylation of downstream signaling molecules. Indeed, AKT, ERK and p38 were also highly phosphorylated in DUSP3-KO mice under CD. Interestingly, under HFD, AKT, ERK, p38 and GSK3 were equally activated regardless of the mice genotype while IR was hyper-phosphorylated in DUSP3-KO livers (Fig. 7e,f). These data suggest IR could be a potential substrate of DUSP3.

### RNA-seq transcriptome analysis and validation

To better investigate the underlying mechanisms behind DUSP3-deletion associated NAFLD progression, we used an unsupervised RNA-seq gene expression profiling of liver extracts from 18 months old DUSP3-KO and WT mice under HFD and CD. PCA identified subgroups of individuals based on diet (Fig. 8a). However, hierarchical clustering of differentially expressed genes (DEGs) identified four clusters of distinct expression patterns that correspond to the different diets and genotypes (Supplementary Fig. S5). To investigate the effect of HFD in the two different genotypes, we generated Fold Change (FC) plots (Fig. 8b,c) that compare DEGs in WT or in DUSP3-KO mice when fed HFD compared to when fed CD. Genes with adjusted p-value < 0.05 and fold change > 1.5 in at least one genotype were plotted. In WT mice, HFD induced an upregulation of 501 genes (right axis) and downregulation of 340 genes (left axis) that were all exclusive to WT mice. In mutant mice only, HFD induced un upregulation of 847 genes (upper axis) and downregulation of 488 genes (lower axis). 416 genes were upregulated and 149 were downregulated in both groups of mice under HFD (Fig. 8b). We next compared the effect of diet on DEGs between WT and mutant mice. As shown in Fig. 8c, 126 genes were upregulated and 197 genes were downregulated in DUSP3-KO mice compared to WT mice under CD. Under HFD, only 52 genes were upregulated and 33 were downregulated in DUSP3 compared to WT mice. Full lists of DEGs for all comparisons are provided in Supplementary Fig. S5 and Supplementary Tables S3 and S4.

Several DUSPs were recently shown to be associated with NAFLD, namely DUSP9(4), DUSP12(6), DUSP14(3) and DUSP26(5). All these DUSPs were described as downregulated in mice livers under HFD. Having RNA-seq data in hands, we checked the expression of all reported DUSPs in WT and DUSP3-KO mice under both CD and HFD (Fig. 8d). Among the 23 DUSPs analyzed, 15 were expressed at different levels in WT mice livers with the highest expression observed for DUSP6, *DUSP3, DUSP11, DUSP1, DUSP16, DUSP12, DUSP22* and *DUSP23*. We did not observe, however, any significant effect of HFD on any of these DUSPs but DUSP3 (Fig. 8d). DUSP3 deletion did not affect the expression of any of these DUSPs (Fig. 8e). To validate the RNA-seq generated data set, we selected 14 genes and evaluated their expression levels using qRT-PCR. Genes selected were part of the IR signaling pathways (*IRS1* and *IRS2*), of lipogenesis and glycogenesis pathways (*FASN, PNPLA3, PPARGC1C, SREBF1, GCK and LEPR*) and DUSPS family members (*DUSP3, DUSP4, DUSP9, DUSP12 and DUSP26*) (Supplementary Fig. S4). Expression levels of all these genes were compatible with RNA-seq generated data.

## Discussion

In this study, using DUSP3-full body knockout mice, we uncovered a critical role of DUSP3 in the development of obesity, NAFLD and associated disorders, and in insulin resistance. A prominent phenotype of DUSP3-KO mice was the development of late-onset obesity and insulin resistance under regular chow diet (CD), a phenotype exacerbated under HFD. Unlike many other animal models available, development of obesity is not accompanied by hyperphagia in DUSP3-KO mice. However, DUSP3-KO mice developed fatty liver, a prevalent complex disease that may lead to severe liver disorders. Indeed, under HFD and DEN, all DUSP3-KO mice developed HCC faster that WT mice. HFD alone was even sufficient for some mutant mice (40%), but not WT, to progress from steatosis to HCC.

NAFLD and associated liver damage in DUSP3-KO mice are evidenced by the increased levels of AST and ALT, of cholesterol and TG, by the increased expression level of fatty acid synthase (FASN), PPARγ and by increased inflammation as suggested by the increased levels of IL6, TNFα and CXCL1 (Supplementary Fig. S4, Supplementary Tables S3 and S4 and data not shown) as well as the infiltration of CD45+ cells in the damaged livers (data not shown). Consistent with this, liver sections of DUSP3-KO mice under both CD or HFD exhibited remarkable increase of lipid accumulation as evidenced by H&E staining. SAF score was also elevated in DUSP3-KO mice compared to WT littermates. Interestingly, DUSP3 expression at protein and mRNA levels was significantly decreased in WT mice under HFD. Such decrease was only observed for DUSP3 but not for any of the other DUSPs family members. This is slightly in contradiction with the published data reporting that DUSP14(3), DUSP9(4), DUSP26(5) and DUSP12(6) were downregulated under HFD. DUSP14 and DUSP9 were even slightly but not significantly increased in response to HFD. For DUSP9, our data are in agreement with Emanuelli et al.^21^. As for the other reports, the discrepancies could be due to differences in the experimental settings including the type of HFD diet, duration of the feeding and animal facility housing conditions. The RNA-seq data also suggest that there are no compensatory mechanisms from other DUSPs in the absence of DUSP3. Indeed, we observed no expression difference between DUSP3-KO and WT in any of the known DUSPs or protein tyrosine phosphatase (PTP) genes.

Interestingly, although young mutant mice were leaner compared to their WT littermates of the same age, insulin levels were significantly higher at all time points analyzed and as early as 3 months after birth. In addition, the insulin receptor was constantly hyperphosphorylated regardless of the type of diet. These data suggest that DUSP3 plays a pivotal role in the insulin signaling pathway.

Insulin is a major hormone that affects directly or indirectly the function of all body tissues, by eliciting a notorious variety of biological responses. Its metabolic actions on the liver, muscle and adipose tissue are responsible for body metabolism and energy storage, and its dysregulation plays an important role in the development of insulin resistance, obesity and type 2 diabetes. Insulin biological actions start by binding to its receptor that belongs to the family of receptors with tyrosine kinase (Tyr) intrinsic activity. This binding generates conformational changes that induce catalytic activation and autophosphorylation of several Tyr residues on the receptor^22^. Autophosphorylated residues are then recognized by different adaptor proteins, which include members of the family of the insulin receptor substrate (IRS), out of which IRS-1 and IRS-2 are the two main substrates and most common intermediates in the initial stage of insulin signal propagation.

Most insulin actions are carried out by activation of two main signaling pathways: the phosphatidylinositol-3-kinase (PI3K)/Akt pathway, responsible for most its metabolic actions, and the mitogen-activated protein kinases/Ras pathway (MAPK/Ras), which regulates gene expression and insulin-associated mitogenic effects^23^. Such important pathways are highly regulated at different levels in order to promote cellular homeostasis. At the receptor level, one of the regulatory mechanisms is governed by PTP1B that dephosphorylate the IR on key Tyr residues that participate in receptor activation and association with adapting proteins. In fact, in PTP1B−/− mice, similarly to DUSP3-KO mice, IR is phosphorylated on Tyr at basal level. However, the observed phosphorylation of IR in PTP1B−/− mice is associated with attenuated Akt and MAPKs activation while the opposite is observed in DUSP3-KO mice. On the other hand, whole-body deletion of PTP1B in mice resulted in increased insulin sensitivity and improved glucose tolerance^24,25^. The animals were found to be lean and resistant to diet-induced obesity^26,27^. The serum cholesterol levels in these animals were found to be lower than in control littermates, even when subjected to HFD for a prolonged period of time^28^. A phenotype that is an opposite of DUSP3-KO mice. These finding suggest that PTP1B and DUSP3 are targeting IR receptor for dephosphorylation on different tyrosine residues with opposite functions.

Other regulatory feedback loops are at Akt and MAPKs levels. Akt activation is under the control of PTEN and SHIP-2 while MAPKs are under the control of several MKPs and DUSPs including DUSP3. It is therefore plausible that DUSP3 is controlling the insulin signaling pathways at the receptor level and at the MAPKs node. Persistent activation of these pathways are well documented mechanisms leading to insulin resistance^23,29^.

Other insulin signaling regulation checkpoints downstream of the recptor are at the level of IRS protein expression and phosphorylation. Levels of IRS2, but not IRS1, are decreased at transcript level in DUSP3-KO mice compared to WT littermates. Deletion of IRS2 in mice causes a progressive development of T2DM. IRS-2−/− mice exhibit mild peripheral insulin resistance and b-cell deficiency at birth but have adequate compensatory insulin secretion for several weeks^30^.

In DUSP3-KO mice, although the insulin level is constantly and significantly high, fasting glucose as well as glucose tolerance are slightly similar to those of WT mice. Glucokinase (GCK) is considered as the primary sensor of glucose not only for regulation of insulin release by pancreatic β-cells, but also for the rest of the cells that contribute to glucose homeostasis in mammals^31^. T2D is associated with decreased level and activity of GCK, an association that provided rationale for trying to find small molecules that activate GCK and thereby improve pharmacological treatment of T2DM^32,33^. We found that GCK levels are significantly increased in DUSP3-KO mice compared to WT littermates regardless of the type of diet. Although we do not know how DUSP3 may regulate GCK expression level, the increased expression of GCK could explain why glucose basal levels and tolerance were similar to those of WT mice.

In summary, although we clearly need to further investigate the possibility that other signaling defects may also contribute to the obese phenotype of the DUSP3-KO animals, the data presented indicate a prominent role for defective IR signaling in the development of obesity and NAFLD in these mice.

## Material and methods

All methods used in this study were carried out in accordance with the relevant guidelines and regulations.

### Mice, high fat diet and liver tumorigenesis

DUSP3 full knockout mice (DUSP3-KO) mice (C57BL/6) were generated and genotyped as previously described^15^. They were backcrossed to C57BL/6 mice for at least ten generations. Homozygous DUSP3−/− mice were obtained by mating DUSP3 ± animals, which were further intercrossed to generate a purebred DUSP3-KO colony. DUSP3+/+ littermates were likewise intercrossed yielding a purebred DUSP3+/+ control colony. Only male mice were used in all experiments. The University of Liege ethical committee approved all protocols under number 1738. The study was carried out in compliance with the ARRIVE guidelines. (https://arriveguidelines.org/). All mice were maintained in filter-topped cages on autoclaved chow diet (CD; composed of 12% fat, 27% protein, and 61% carbohydrates based on caloric content, RM3, Special Diet Service, United Kingdom) or high-fat-diet (HFD; composed of 42% fat, 15% protein, and 43% carbohydrates based on caloric content; sniff^®^ TD.88137, Soest, Germany) and water. All animals were housed in specific pathogen free conditions and maintained in a temperature and light (12 h light/dark cycle) controlled animal facility. Food consumption was evaluated during a 27-weeks period by weighting the food every week.

For chemical induction of hepatocarcinogenesis, 14 days postnatal mice were intraperitoneally injected with diethylnitrosamine (DEN; 25 mg/kg, Sigma-Aldricht, St Louis, Missouri, United States), then weaned at 5 weeks and maintained on CD. Four weeks after the injection, mice were separated into two dietary groups and fed either CD or HFD until sacrifice. Weight of the animals was evaluated every week for the entire duration of the experiment. Animals were sacrificed by cervical dislocation at week 24 or 32 after DEN injection. All soft tissues were harvested and weighted the day of sacrifice.

Subcutaneous, epididymal and brown fat were dissected, weighed and paraffin embedded for hematoxylin and eosin (H&E) staining.

Livers and hepatic tumors were pictured and harvested. Livers were separated into individual lobes. Externally visible tumors (> 1 mm) were counted and their size measured with a caliper. Large lobes were embedded in paraffin.

### Immunohistochemistry

Livers were embedded in paraffin, sectioned (5 µm) and stained with H&E to evaluate morphology. Sirius Red stain was used to determine fibrosis and a reticulin stain (Foot stain) to evaluate hepatic trabeculae architecture. Liver histology was evaluated by a pathologist. For each specimen, SAF score^34^ (steatosis, activity, fibrosis) summarizing the main histological lesions was defined. Briefly, four histological features were semi-quantitatively assessed: steatosis (0–3), lobular inflammation (0–3), hepatocellular ballooning (0–2), and fibrosis (0–4). Scores of the four features were added to give the SAF score. This score was used to compare liver damages and inflammation between DUSP3-KO and control mice in all experimental conditions. Hepatic nodules were categorized into dysplastic nodules and hepatocellular carcinoma.

Epididymal fat was embedded in paraffin, sectioned and stained with H&E. Adipocytes size was assessed using ImageJ software^35^ by detecting the edge of the adipocytes and measuring the area of cells bigger that 300 mm^2^ and with a circularity between 0.3 and 1. Three fields per section per mouse were used. All stained sections were scanned using a NDP NanoZoomer Digital Pathology scanner (Hamamatsu, Shizuoka, Japan).

### MicroCT imaging

Body composition of CD and HFD fed DUSP-3 KO and WT mice was assessed by X-ray computed tomography (CT) imaging using an eXplore 120 micro-CT (Gamma Medica, USA/GE Healthcare, United kingdom) with a customized protocol. Fat and lean volumes were assessed by semi-automated segmentation procedure using PMOD 3.6 software (PMOD Technologies, Zurich, Switzerland; RRID:SCR_016547). Fat and total body volumes were calculated as % Fat mass = Fat Volume × 100/Total Body Volume and % Lean and bone mass = [Total Body Volume − Fat Volume] × 100/Total Body Volume.

### Glucose tolerance assay

Mice were starved for 6 h. Blood was drawn from a tail nick before and at the indicated time points after *per os* administration of glucose (2 g/kg body weight). Blood glucose was instantly measured using a Contour XT glucometer (Ascencia Diabetes Care, Machelen, Belgium).

### HOMA

Insulin sensitivity was estimated using the Homeostatic Model Assessment of Insulin Resistance (HOMA-IR) method, calculated using the formula: basal insulin (mU/L) × basal glucose (mg/dL)/405 as previously reported^36^.

### Serological and biochemical analyses

Blood was collected from the retro-orbital plexus using a capillary on anesthetized mice right before euthanasia. Serum was separated after clotting by centrifuging the blood at 2300*g* for 10 min. ALT, AST, triglycerides, LDL, HDL and total cholesterol levels in serum were measured using AU480 chemistry analyzer (Beckman Coulter, Brea, California, United States). Plasma insulin concentrations were measured by ELISA (Mercodia, Uppsala, Sweden).

### Tissue homogenization and Western blot

Liver samples were homogenized using a tissue lyser in RIPA buffer and protein concentration was measured using the colorimetric Bradford reagent (Bio-Rad, Nazareth, Belgium). Equal amounts of proteins were run on SDS-PAGE gel and transferred to Hybond-nitrocellulose membranes. Membranes were incubated with corresponding primary antibodies overnight at 4 °C. After incubation with HRP conjugated secondary antibodies, blots were developed by enhanced chemiluminescence (Thermo Scientific, Waltham, Massachusetts, United States) according to manufacturer’s instructions. Antibodies are listed in Supplementary Table 1.

### RNAseq and qRT-PCR

RNAsequencing (RNAseq) and qRT-PCR were performed on RNA extracted from individual livers of CD and HFD fed WT and DUSP3-KO mice of 18 months old and according to the protocols in the Supplementary Information. Primers used for qRT-PCR are listed in Supplementary Table S2.

### Statistical analysis

Statistical analysis and graphics were performed using GraphPad prism 7. The values plotted represent the mean of the biological replicates ± the standard deviation (SD). Mann–Whitney tests were employed when two groups were compared. P-value less than 0.05 was considered as significant. *p < 0.05, **p < 0.01, ***p < 0.001. For high throughput sequencing, statistical significance was assessed using the q-value. One-way ANOVA was used to compare means between groups and a Bonferroni correction was applied to account for multiple testing.

## Supplementary Information

Supplementary Information.
