# Supplementary material for: Dual-specificity phosphatase 3 deletion promotes obesity, non-alcoholic steatohepatitis and hepatocellular carcinoma
Source: Sci Rep. 2021 Mar 12;11:5817. doi: 10.1038/s41598-021-85089-6 (PMC7954796; doi:10.1038/s41598-021-85089-6)
Supplement: Supplementary file 1 — Supplementary Information. [file 41598_2021_85089_MOESM1_ESM.pdf]

Dual-Specificity Phosphatase 3 deletion promotes obesity, non-alcoholic steatohepatitis and hepatocellular carcinoma

Sophie Jacques, Arash Arjomand, Hélène Perée, Patrick Collins, Alice Mayer, Arnaud Lavergne, Marie Wéry, Myriam Mni, Alexandre Hego, Virginie Thuillier, Guillaume Beckers, Mohamed Ali Bahri, Alain Plenevaux, Emmanuel Di Valentin, Cécile Oury, Michel Moutschen, Philippe Delvenne, Nicolas Paquot, Souad Rahmouni

Supplementary material

Supplementary material and methods

Antibodies

Supplementary table S1. Antibodies for immunoblot analysis.

Target	Reference	Firm
DUSP3	8889	Santacruz
Phospho-p44/42 MAPK (ERK1/2) (Thr202/Tyr204)	9101	Cell Signaling
P44/42 MAPK (ERK1/2)	9102	Cell Signaling
Phosphor-Akt (Ser473)	9271	Cell Signaling
Akt	9272	Cell Signaling
Phospho-IGF-1 Receptor β (Tyr1135/1136)/Insulin Receptor β (Tyr1150/1151)	3024	Cell Signaling
Insulin Receptor β (4B8)	3025	Cell Signaling
Phospho-GSK-3α/β (Ser21/9)	9331	Cell Signaling
GSK-3α/β	7291	Santacruz
Phospho-p38 MAPK (Thr180/Tyr182)	9215	Cell Signaling
P38 MAPK	9212	Cell Signaling
Actin	SAB5500001	Sigma-Aldrich

qRT-PCR

2µg of liver total RNA were reverse-transcribed to obtain cDNA (RevertAid H Minus First Strand cDNA Synthesis Kit, ThermoFisher Scientific, Waltham, Massachusetts, United States). Each cDNA was analyzed, in triplicate, with the ABsolute Blue QPCR Mix, SYBR Green, ROX (ThermoFisher Scientific, Waltham, Massachusetts, United States) in the CFX384 Touch Real-Time PCR Detection System (BioRad, Nazareth, Belgium) The results were normalized to Ribosomal Protein S9 (RPS9). The relative mRNA expression levels were calculated using the 2-ΔCt method. Used primers are presented in Supplementary Table 2 (IDT, Coralville, Iowa, United States).

Supplementary table S2. Primers used for qRT-PCR.

Gene	FW	RV
DUSP3	GGGTGATGCCCAGTTTCT	GATCTCAACGACCTGCTCTC
DUSP4	CACGGACATCTGCCTGCTTAAA	GGCCAGGGCCTTGGTTT
DUSP9	AGAAGGCTACCCAGCATACTA	GCACTGGGCTAGACATTGAG
DUSP12	TCTCTTGTGAGCAAAGGCTATT	CTTCGCTGTTGACCCAACTA
DUSP26	TAACCATGCTGACGAAGTCTG	TTGAGCACGTGGGTGATG
FASN	TGGCTGTGTATTCCAGTTGTAG	CTCAAGATGAAGGTGGCAGAG
PNPLA3	TCTCACTCACCAGGGTGTC	GGGAATGAAGCAGGAACACA
SREBF1	CATGCCCTCCATAGACACATC	AGAAGCTCAAGCAGGAGAAC
IRS2	GTCCAGGCACTGGAGCTTT	GCTGGTAGCGCTTCACTCTT
LEPR	GACTTGCAGATGGTCACCCA	TGGGCTCAGACGTAGGATGA
GCK	CAACTGGACCAAGGGCTTCAA	TGTGGCCACCGTGTCATTC
RPS9	CACGGAGACCCTTCGAGA	GTAAACTTGACCCTCCAAACCT

RNA-sequencing and analysis

Total RNA was extracted from 20mg of frozen liver using the Maxwell® 16 LEV simplyRNA Tissue Kit (Promega, Madison, Wisconsin, United States), according to manufacturer's instruction. RNA quantity was assessed using a spectrophotometer (NanoDrop Technologies, Wilmington, Delaware, United States). Total RNA integrity was evaluated by QIAxcel Advanced technology (Qiagen, Hilden, Germany). RNA libraries were prepared using Truseq stranded mRNA sample prep kit from Illumina (San Diego, California, United States), based on polyA selection of mRNA. cDNAs fragments were sequenced using the Illumina NextSeq500 (San Diego, California, United States). . Biological triplicates were performed for all the conditions.

Raw reads were quality controlled using FastQC (version 0.11.5) and aligned to the Ensembl mouse reference genome GRCm38 (release 97) using STAR (version 7.1a)(1). Gene expression quantification was obtained with the --quantMode GeneCounts STAR option. Normalization and differential expression analysis were performed using the DESeq2 R package(2), with the apeglm method for effect size shrinkage(3). Genes with a q value below 0.05 and a fold change above 1.5 were considered differentially expressed. Heatmaps and fold change plots were performed using ggplot2 R package.

For GSEA analysis of RNA-sequencing data, GSEA software available on the GSEA-Broad Institute website was used. Mouse Ensembl gene identifiers were converted to human orthologues. Normalized gene expression data set was run against a library of 50 curated gene sets for hallmark pathways (MSigDB v7.1). The statistical significance (False Discovery Rate (FDR) q-value) was estimated by running 1000 gene set permutations.

MicroCT imaging

Body composition of CD and HFD fed DUSP-3 KO and WT mice was assessed by X-ray computed tomography (CT) imaging. CT scans were acquired using an eXplore 120 micro-CT (Gamma Medica, USA/GE Healthcare, UK) with a customized protocol (70 kV, 0.512 mAs, 360 views over 360°, continuous rotation) provided by the manufacturer (4),(5). Mice were anesthetized with isoflurane. Respiratory rate and body temperature were continuously monitored during the procedure. All micro-CT images were reconstructed using the Feldkamp's filtered back-projection algorithm with a cutoff at the Nyquist frequency to obtain a 3D volume with an isotropic voxel size of 100 µm. To assess adipose tissue CT signal intensity, CT scans of different freshly harvested fat types (epidydimal white fat, subcutaneous white fat, adrenal white fat and brown fat) were acquired.

Fat and lean volumes were assessed by semi-automated segmentation procedure using PMOD 3.6 software (PMOD Technologies, Zurich, Switzerland; RRID:SCR_016547). Briefly, an intensity threshold range (-280 to -160 HU (Hounsfield unit)) for fat was obtained based on the images of the ex-vivo harvested fat. In-vivo CT image of the whole mouse was sent to the PMOD automated segmentation to extract the fat part. Based on the mean signal intensity (threshold for bone: 250 HU), extracted on a spherical region of interest placed on the bone, binary mask of the bone was extracted using automated segmentation method implemented in PMOD. A total body mask was also generated using a range of signal intensities containing fat, bone, and muscle. Knowing the voxel size and the number of voxels in the obtained masks, the fat, bone and total body volumes were calculated.

The % of Fat mass was calculated as follow:

% Fat mass = Fat Volume *100/ Total Body Volume

% Lean and bone mass= [Total Body Volume – Fat Volume] *100/ Total Body Volume

Supplementary results

Supplementary table S3. Differentially expressed genes between WT and DUSP3-KO mice under CD.

Gene name	Base Mean	log2 Fold Change	lfcSE	pvalue	padj
BC018473	61,08668	8,681199	2,623081	1,43E-10	9,47E-08
Gm28438	10,46834	5,403573	1,343156	2,83E-06	0,000464
AC121973.1	1066,416	3,894238	0,541599	3,05E-14	6,35E-11
Slc13a4	16,96862	3,442846	1,191334	0,00019	0,012367
Slc22a29	51,80212	2,608627	0,570525	2,21E-07	5,56E-05
Tnfaip8l3	16,59083	2,528087	0,833577	0,000185	0,012165
Sult2a7	497,7114	2,485481	1,026678	0,000384	0,019494
Gck	2650,667	2,353243	0,391146	7,79E-11	5,59E-08
Lrtm1	122,5058	2,283397	0,810075	0,000144	0,010095
Tsku	1731,861	2,224796	0,501571	3,69E-07	8,53E-05
Gprc5b	127,992	2,149273	0,657558	3,75E-05	0,003624
Adgrv1	430,7787	2,100606	0,548934	4,93E-06	0,000733
Gm36041	199,7098	1,993876	0,67525	0,000102	0,007779
Fam25c	599,7406	1,960855	0,617637	5,14E-05	0,004602
A530020G20 Rik	44,81259	1,958284	0,787831	0,000405	0,020118
Aatk	220,9329	1,932801	0,304295	1,04E-11	1,08E-08
B430212C06 Rik	69,20869	1,843239	0,704276	0,000275	0,015813
Cxcl1	350,9636	1,770165	0,647952	0,000197	0,012596
Slc22a27	159,6481	1,742171	0,753385	0,000569	0,024624
Twist1	10,58552	1,731414	0,832107	0,001461	0,04746
Angptl8	1912,465	1,698778	0,448251	5,9E-06	0,00082
Gm45941	23,62497	1,666457	0,601722	0,000222	0,013733
Saa3	498,2451	1,512821	0,605867	0,000383	0,019494
Adora1	667,4911	1,509423	0,285775	6,06E-09	2,76E-06
Tsc22d1	1585,504	1,413191	0,305869	1,89E-07	5,01E-05
Fcor	69,87633	1,407807	0,584358	0,000498	0,022603
Leap2	4474,507	1,394632	0,606791	0,000603	0,025561
Eda2r	36,15298	1,375382	0,588245	0,000625	0,026175
Gm3219	28,94414	1,363962	0,4177	4,81E-05	0,00438
Gm11967	60,64426	1,360183	0,507707	0,000263	0,015435
Ppp1r3c	1673,915	1,334909	0,330506	2,41E-06	0,000413
S1pr1	1022,217	1,322611	0,271081	5,07E-08	1,68E-05
Mapk4	34,48571	1,317896	0,45069	0,000137	0,009854

2510016D11 Rik	26,63388	1,303332	0,520078	0,000429	0,020902
Man2c1os	28,50406	1,2732	0,550417	0,00068	0,027914
9030619P08 Rik	207,0271	1,269385	0,374795	2,88E-05	0,002957
Wdfy1	442,6566	1,252201	0,191888	3,45E-12	5,04E-09
Bcl7c	234,3844	1,241851	0,219432	7,65E-10	4,59E-07
Syt3	109,0069	1,239582	0,337453	1,04E-05	0,001277
Nim1k	44,60683	1,231837	0,500392	0,00047	0,022038
Extl1	194,7231	1,192456	0,56434	0,000999	0,036333
Lgals1	1103,921	1,120122	0,438019	0,000356	0,018902
Orai1	222,7327	1,108715	0,245639	3,17E-07	7,85E-05
Brca2	55,57081	1,099428	0,371942	0,000129	0,00941
Acot11	192,8185	1,094551	0,376872	0,000145	0,010095
Cry1	153,1225	1,086219	0,307373	1,82E-05	0,002013
Wdr91	432,0544	1,067826	0,220585	6,79E-08	2,11E-05
Snai2	98,18259	1,05691	0,293287	1,47E-05	0,001721
Sipa1l2	91,54609	0,992331	0,382032	0,00036	0,018928
Lin9	55,96553	0,989077	0,414051	0,000624	0,026175
Saa4	3989,58	0,988811	0,445849	0,00095	0,035337
Cebpe	134,7867	0,981576	0,410319	0,000602	0,025561
Ubtd1	168,209	0,954629	0,298716	6,26E-05	0,005437
Ccdc85c	328,1395	0,931646	0,147417	1,54E-11	1,5E-08
Zfp385a	335,7378	0,930775	0,248858	8,99E-06	0,001152
Myorg	693,8881	0,927971	0,244246	7,42E-06	0,000975
Cyp2j9	397,3673	0,913251	0,312523	0,00015	0,010287
Abcb9	56,33894	0,913154	0,350179	0,000382	0,019494
Tjp3	364,5132	0,910429	0,297388	0,0001	0,007722
Pdzrn3	81,3333	0,905305	0,424037	0,001126	0,039484
F830016B08 Rik	156,2251	0,901901	0,455235	0,00151	0,048509
Pigyl	347,1542	0,881351	0,351087	0,000484	0,02232
Herc3	163,3345	0,878096	0,270027	5,37E-05	0,004773
Slc6a9	873,1387	0,874546	0,298612	0,00015	0,010299
Gm42604	62,30693	0,869012	0,357272	0,000592	0,025328
Shf	224,9846	0,866603	0,233086	1,03E-05	0,001277
Ahr	891,7482	0,862652	0,186264	2,02E-07	5,26E-05
D630039A03 Rik	237,8987	0,853386	0,332007	0,000414	0,02035
Col5a3	1030,653	0,84714	0,280764	0,000119	0,008773
D930048N14 Rik	180,4646	0,840365	0,353716	0,000681	0,027914
Prxl2b	302,3913	0,837226	0,353002	0,000708	0,028431
A230050P20 Rik	2141,484	0,828651	0,282941	0,000168	0,011291
Tert	101,2178	0,807315	0,374865	0,001169	0,04046

Nup93	147,2572	0,803338	0,313092	0,000437	0,021122
Rcc2	764,6012	0,797514	0,184873	9,32E-07	0,000184
Glud1	20936,96	0,797253	0,246293	6,04E-05	0,00531
1810058I24Rik	2271,99	0,794508	0,19637	2,9E-06	0,00047
Pgp	422,1214	0,788707	0,159699	5,36E-08	1,7E-05
Inhbc	1798,292	0,78491	0,250351	8,37E-05	0,006859
Stk38l	360,2986	0,7709	0,259559	0,000145	0,010095
Mid1ip1	773,1517	0,768517	0,286013	0,000326	0,017759
Wdr6	425,1099	0,768061	0,196553	5,52E-06	0,000796
Rps6ka1	377,2825	0,76782	0,20726	1,15E-05	0,00137
Gm15446	60,78648	0,766327	0,330095	0,000841	0,032528
Bax	503,319	0,759068	0,172704	6,61E-07	0,00014
Tle3	403,3288	0,75709	0,272432	0,000254	0,015019
Gm12909	276,3702	0,75334	0,34944	0,001217	0,041455
Atp23	83,51953	0,752598	0,348285	0,001194	0,040966
Chd9	337,7558	0,752394	0,184244	2,58E-06	0,000438
Asap3	201,3774	0,752386	0,277546	0,000312	0,017377
Aars2	276,7543	0,744104	0,200334	1,13E-05	0,001368
Rtn4rl1	417,5824	0,740634	0,280426	0,000385	0,019494
Dock4	497,4657	0,736862	0,216345	3,58E-05	0,003483
Ssx2ip	196,8453	0,731867	0,220971	5,01E-05	0,004536
Gpr135	67,06414	0,728081	0,322648	0,001019	0,036884
Cited2	306,059	0,727096	0,266346	0,000304	0,017054
Aox3	6062,398	0,718071	0,341568	0,001414	0,046339
Ldlrad3	123,9164	0,717327	0,298422	0,000719	0,028647
Ttc7	989,8676	0,715343	0,208972	3,44E-05	0,00339
Slc25a23	3268,101	0,70706	0,311911	0,001003	0,036394
Rpusd3	255,2211	0,703633	0,240025	0,000175	0,011725
Tmc6	314,1529	0,702302	0,284414	0,000624	0,026175
Pllp	404,744	0,699165	0,185791	1,01E-05	0,001264
Ptprf	2273,344	0,691463	0,193314	2,04E-05	0,002201
Kifc2	169,6803	0,690805	0,306641	0,00106	0,037596
Pnkd	1871,871	0,690659	0,280974	0,000689	0,028064
Gsap	657,8006	0,685217	0,242193	0,000245	0,014664
Mug2	7439,878	0,685105	0,264845	0,00048	0,02232
Slc36a1	595,9387	0,682509	0,210528	6,71E-05	0,005759
Fbxw9	879,5844	0,681367	0,245178	0,000281	0,016074
Gpsm2	308,2671	0,680085	0,173467	5,47E-06	0,000796
Pias3	220,5459	0,674491	0,199559	4,24E-05	0,004044
Cars	618,0213	0,673058	0,257433	0,000446	0,021164
Polr2i	561,5536	0,671855	0,262443	0,000517	0,02305
Lrrc42	572,5879	0,66709	0,225289	0,000167	0,011291
Atp13a2	307,7564	0,658681	0,293038	0,00112	0,039373
Igsf8	211,0695	0,645859	0,290439	0,001153	0,040143

Dusp28	211,8871	0,640692	0,231335	0,000307	0,017152
Svil	412,1398	0,640403	0,294218	0,001339	0,044802
Tsen54	176,5685	0,63977	0,226922	0,000268	0,015556
Slc9a6	409,7318	0,636858	0,225914	0,000269	0,015556
Rasl11b	61,97577	0,634773	0,279671	0,001144	0,040008
Nectin1	1235,408	0,630397	0,227812	0,000343	0,018396
Rxrg	160,3029	0,628702	0,272628	0,00105	0,03736
Pold2	164,8219	0,625812	0,21925	0,000244	0,014625
Rpp21	195,4837	0,608352	0,235234	0,000535	0,023663
Tcea3	1122,757	0,607874	0,228995	0,000445	0,021164
Klhl22	357,2525	0,606998	0,156199	6,99E-06	0,000935
Dop1b	653,1866	0,606645	0,241904	0,000651	0,027057
Celsr1	271,3317	0,602876	0,264909	0,00115	0,04013
Fgfr4	988,1454	0,600057	0,190177	0,000101	0,007726
4933439C10 Rik	161,4305	0,593134	0,214065	0,000333	0,018042
Fuk	189,1037	0,591084	0,249289	0,000941	0,035142
Acbd4	1221,292	0,588261	0,249092	0,00098	0,036167
Prkcz	324,8077	-0,58157	0,209225	0,000326	0,017759
Zfp750	238,1661	-0,58441	0,259419	0,001238	0,042072
Gtf2b	535,8008	-0,58718	0,186398	0,000104	0,007894
Tec	543,9289	-0,58732	0,162144	1,97E-05	0,002164
Hnrnpa1	1342,822	-0,58936	0,199119	0,000186	0,012193
Tbrg1	1237,256	-0,58952	0,207952	0,00027	0,015556
Sri	846,9037	-0,59061	0,174776	4,66E-05	0,004316
Chp1	7157,596	-0,59341	0,1948	0,00014	0,009976
Krt18	5352,656	-0,59584	0,273482	0,001429	0,046711
Dlst	5247,41	-0,5983	0,131507	4,02E-07	9,15E-05
Rbm4b	403,5569	-0,60511	0,243991	0,000691	0,028088
Uspl1	506,4438	-0,60606	0,195642	0,000115	0,008567
Tnfaip8l1	941,1454	-0,60789	0,263447	0,001048	0,03736
Gpt2	15174,01	-0,61095	0,226925	0,000389	0,019608
Daglb	749,7578	-0,61352	0,202937	0,000152	0,010382
Hps4	293,235	-0,61422	0,204433	0,000156	0,010591
Pdlim1	484,3492	-0,61737	0,237472	0,000508	0,022973
Rpl3	5245,907	-0,62044	0,276147	0,001171	0,04046
Trib1	1334,42	-0,63301	0,259806	0,00071	0,028434
Tmed4	2362,873	-0,63353	0,115572	2,99E-09	1,56E-06
Rbpms	1096,41	-0,63416	0,263915	0,000793	0,031103
Fam169b	512,9445	-0,65755	0,254453	0,000482	0,02232
Hspd1	6138,804	-0,65866	0,155921	1,78E-06	0,000321
Mafk	331,1919	-0,66223	0,305654	0,001304	0,04381
Acox1	61105,58	-0,6668	0,211186	8,7E-05	0,007013
Pnpla7	5475,373	-0,66833	0,261342	0,000568	0,024624
Odc1	954,7727	-0,67347	0,220649	0,000122	0,008943

Kif21a	1304,89	-0,67676	0,177688	8,38E-06	0,001091
Ppp2r2d	1065,13	-0,67833	0,189325	1,99E-05	0,002167
Mapk1ip1	268,1825	-0,68011	0,182102	1,12E-05	0,001358
Parp16	618,9565	-0,68041	0,302879	0,001036	0,037196
Catsper2	81,30797	-0,68082	0,309377	0,0012	0,040993
Lgals8	3858,14	-0,68175	0,128526	7,82E-09	3,26E-06
Midn	949,37	-0,68281	0,257974	0,000401	0,020118
Ripk4	118,0489	-0,68943	0,27289	0,000551	0,024215
Rfx4	166,4253	-0,6915	0,217488	8,1E-05	0,006713
Arrdc4	366,8634	-0,69258	0,245474	0,000242	0,01458
Arsg	1110,235	-0,6974	0,326578	0,001333	0,044691
Npc1	2844,732	-0,70057	0,242248	0,000192	0,0124
Palmd	829,4878	-0,72429	0,260675	0,000264	0,015435
Amigo2	263,8222	-0,73694	0,257518	0,000205	0,012878
Hykk	2031,539	-0,74017	0,286581	0,000438	0,021122
Rsad1	353,725	-0,74518	0,212583	2,45E-05	0,002607
Grpel2	1558,669	-0,75013	0,208791	1,74E-05	0,001949
Grb14	1455,011	-0,75152	0,172747	7,91E-07	0,000162
Tnfrsf1b	817,7566	-0,75271	0,271027	0,000247	0,014678
Pank1	6147,412	-0,75305	0,217006	2,56E-05	0,002672
Mycl	214,263	-0,75373	0,284757	0,000366	0,019145
Arl13b	164,5136	-0,75616	0,301252	0,000521	0,023081
Erbb3	2609,092	-0,75668	0,192147	4,57E-06	0,000693
Fads2	16996,98	-0,76125	0,221907	3,13E-05	0,00315
Tra2b	698,7221	-0,76346	0,177081	8,91E-07	0,000181
Wrnip1	1513,371	-0,767	0,173099	5,26E-07	0,000113
Fh1	6279,54	-0,77196	0,364889	0,001302	0,04381
Reps1	518,6072	-0,77199	0,180617	1,09E-06	0,000209
Josd2	853,5204	-0,78004	0,251714	9,34E-05	0,007406
Mat1a	117299,4	-0,78093	0,228629	3,21E-05	0,003209
Slc25a32	664,1264	-0,78831	0,251168	8,28E-05	0,006826
Tor1b	1659,597	-0,79817	0,192062	1,77E-06	0,000321
Tpm4	616,748	-0,80572	0,308551	0,000398	0,020025
Epha2	589,435	-0,80847	0,354958	0,000866	0,033374
Hsp90aa1	1795,178	-0,8242	0,412269	0,001505	0,048509
Ifrd1	491,8712	-0,83117	0,198533	1,53E-06	0,000282
Glyctk	5443,891	-0,8475	0,29786	0,000191	0,01237
Tspan31	9213,027	-0,85506	0,177196	7,29E-08	2,21E-05
Pop1	106,3318	-0,86217	0,33946	0,000439	0,021122
Hmgn2	226,8144	-0,86217	0,375944	0,000807	0,031385
Slc22a5	971,5006	-0,86283	0,321944	0,0003	0,01689
Arhgef37	154,6442	-0,86812	0,357774	0,000583	0,025097
Cth	11311,29	-0,86955	0,440426	0,00139	0,045865
Hsd17b13	11873,09	-0,87046	0,265646	5,07E-05	0,004564
Hspa8	16381,2	-0,87053	0,222815	4,71E-06	0,000708

Scamp1	1806,738	-0,87755	0,157591	1,43E-09	8,04E-07
Pnpla2	2011,608	-0,88857	0,281757	7,35E-05	0,006193
Cln8	809,6995	-0,88987	0,209557	1,1E-06	0,000209
Agxt	6331,707	-0,90005	0,306112	0,000137	0,009854
Tgfbr3l	55,71719	-0,91384	0,382552	0,000638	0,026565
Acacb	1593,952	-0,91484	0,351468	0,000361	0,018928
4933431K14 Rik	43,39567	-0,91681	0,416777	0,00099	0,036167
Slc25a22	6333,805	-0,91785	0,330754	0,000221	0,013733
Fbf1	291,5309	-0,92374	0,357191	0,000374	0,019298
Cystm1	265,0839	-0,92542	0,353295	0,000344	0,018396
Apbb3	432,6853	-0,93259	0,310954	0,000115	0,008567
Rab30	857,6835	-0,93566	0,355309	0,000321	0,017673
Angptl4	2985,981	-0,9501	0,409427	0,000684	0,027942
Pfkfb1	767,8397	-0,95675	0,29008	4,3E-05	0,004071
Setd7	145,2742	-0,96679	0,374097	0,000369	0,019172
Pck2	120,4039	-0,96804	0,384751	0,000445	0,021164
Plk3	2025,291	-0,97075	0,42319	0,00073	0,028946
Ppp1r9a	296,1184	-0,97256	0,384083	0,000415	0,02035
Rhpn2	523,8625	-0,97422	0,365513	0,000293	0,016633
Elovl2	12715,88	-0,97746	0,217884	3,63E-07	8,53E-05
Ppargc1a	250,2223	-0,98317	0,336565	0,000141	0,010033
Myh10	252,1581	-0,98371	0,276793	1,72E-05	0,001949
Atg16l2	423,2445	-0,98775	0,387656	0,000406	0,020118
Mppe1	425,312	-0,98947	0,265379	9,08E-06	0,001152
Tmtc2	263,9885	-0,99504	0,293612	3,1E-05	0,00315
Nceh1	1179,487	-0,99759	0,264093	7,25E-06	0,000962
Lgals4	960,3499	-0,99939	0,28158	1,72E-05	0,001949
Elovl6	1375,307	-1,0043	0,373438	0,00027	0,015556
Slc35e2	1482,959	-1,00542	0,238321	1,21E-06	0,000226
Armcx3	59,45779	-1,01052	0,402112	0,000444	0,021164
Slc20a1	1202,647	-1,01254	0,332905	9,57E-05	0,007547
Spred2	164,698	-1,0164	0,322581	6,76E-05	0,005769
Sgce	158,0384	-1,02355	0,297118	2,55E-05	0,002672
Elovl5	18388,37	-1,02362	0,304122	3,13E-05	0,00315
Apom	4688,781	-1,03574	0,209059	3,52E-08	1,28E-05
Hsf2bp	106,0766	-1,03616	0,440429	0,000635	0,026537
Abhd1	125,5966	-1,03629	0,257626	2,73E-06	0,000453
Fmo2	280,7485	-1,04228	0,25571	2,14E-06	0,000376
Gm30122	96,19339	-1,04735	0,356608	0,000132	0,009585
Chrna2	277,9384	-1,05609	0,349592	9,96E-05	0,007722
Slc25a47	22998,55	-1,05985	0,195552	3,39E-09	1,7E-06
Cpt1a	11910,49	-1,07807	0,187433	4,35E-10	2,76E-07
Slc25a30	983,5885	-1,08895	0,366465	0,000113	0,008483
Sorbs3	1135,035	-1,10139	0,295764	9,08E-06	0,001152

Il17rb	291,9299	-1,10632	0,2514	5,15E-07	0,000112
Rapgef4	2567,169	-1,10766	0,351236	6,2E-05	0,005414
Mknk2	2769,843	-1,12963	0,294921	5,62E-06	0,0008
Zc3h12d	100,8982	-1,1303	0,473803	0,000566	0,024624
St3gal5	3198,145	-1,13749	0,266786	9,76E-07	0,00019
Gm32063	198,8228	-1,14122	0,45534	0,000409	0,020212
Hspb1	276,2499	-1,14315	0,395539	0,000144	0,010095
9330159M07 Rik	47,8234	-1,14361	0,509844	0,000782	0,03088
Plin2	20226,49	-1,15518	0,215095	3,5E-09	1,7E-06
Slc16a5	481,5501	-1,15898	0,245713	1,15E-07	3,22E-05
Rogdi	504,3495	-1,17202	0,296245	3,39E-06	0,000531
Itih5	242,2679	-1,17209	0,327475	1,47E-05	0,001721
Slc25a34	230,8459	-1,18706	0,249202	8,9E-08	2,6E-05
Tent5c	211,9448	-1,18838	0,54801	0,000867	0,033374
Map3k5	816,3852	-1,19074	0,257949	1,81E-07	4,88E-05
Tagln	213,4756	-1,20343	0,46596	0,000324	0,017751
Ten1	163,2439	-1,20433	0,312207	5,15E-06	0,000759
Enc1	346,6626	-1,21973	0,376822	4,71E-05	0,004321
Tacc2	614,4005	-1,22901	0,323839	6,29E-06	0,000865
C9orf72	501,3943	-1,22923	0,312565	3,66E-06	0,000568
Igfbp2	10998,02	-1,23357	0,575717	0,000887	0,033771
Odf3b	133,9352	-1,25299	0,396101	6,02E-05	0,00531
Btbd19	56,29831	-1,25622	0,491784	0,000359	0,018928
Irs2	534,8822	-1,27567	0,357203	1,36E-05	0,001618
Lad1	167,4237	-1,27578	0,411666	7,33E-05	0,006193
1810055G02 Rik	940,4041	-1,2836	0,320317	2,63E-06	0,000441
Pctp	4008,723	-1,29354	0,282894	2,2E-07	5,56E-05
Slco1a4	1419,517	-1,31385	0,372144	1,61E-05	0,001848
Sik1	745,8529	-1,31548	0,519647	0,000341	0,018396
Retreg1	2201,456	-1,32409	0,234465	7,87E-10	4,59E-07
Ccn2	534,8743	-1,33252	0,539388	0,000416	0,02035
Tra2a	728,7159	-1,33354	0,219197	5,64E-11	4,62E-08
Gm33447	24,77386	-1,34049	0,587611	0,000702	0,028261
Abhd2	4200,483	-1,34416	0,208734	5,95E-12	7,89E-09
Fgfr1	208,2057	-1,37419	0,511548	0,000237	0,01436
Frmd4b	1114,108	-1,37439	0,360472	5,67E-06	0,0008
Rdh16f2	2053,347	-1,38444	0,365445	5,7E-06	0,0008
Pgm3	676,5948	-1,38498	0,205787	8,33E-13	1,35E-09
Lpin2	7582,94	-1,42009	0,279673	1,72E-08	6,42E-06
Cdc42ep5	100,0224	-1,44459	0,47046	7,69E-05	0,006407
Slc2a5	220,8274	-1,47887	0,506207	0,000119	0,008773
Hhipl2	59,28946	-1,48259	0,53936	0,000202	0,012798
Cyp2d41-ps	58,95505	-1,48319	0,330906	3,41E-07	8,29E-05

Slc25a27	35,14539	-1,4934	0,500192	0,000108	0,008165
Cyp4a32	759,6728	-1,50319	0,281496	4,19E-09	1,97E-06
Cidec	1961,58	-1,50659	0,341869	4,49E-07	9,93E-05
Serpinb6b	169,0611	-1,50906	0,56221	0,000235	0,014301
Gstp2	166,7451	-1,56769	0,665727	0,000511	0,022973
Nlrp12	2506,553	-1,65427	0,318488	8,95E-09	3,63E-06
Gm34654	97,99884	-1,65497	0,48337	2,28E-05	0,002447
Cables1	57,6669	-1,66498	0,355692	1,36E-07	3,75E-05
Ncmap	31,2672	-1,67374	0,532794	6,47E-05	0,005583
Lamb3	267,927	-1,69611	0,264987	7,54E-12	9,16E-09
Sel1l3	412,7181	-1,69969	0,350642	5,01E-08	1,68E-05
Gm35696	128,115	-1,72751	0,65831	0,000254	0,015019
Tbc1d8	451,6229	-1,73702	0,389115	3,61E-07	8,53E-05
Gys2	5041,325	-1,75131	0,31744	1,54E-09	8,3E-07
Gm15663	13,55922	-1,75654	0,689756	0,000382	0,019494
Dusp3	990,497	-1,84107	0,127817	2,33E-48	3,39E-44
Cntrl	739,5584	-1,85414	0,246881	2,68E-15	7,81E-12
Gm44509	16,61769	-1,85989	0,681087	0,000344	0,018396
Gm32540	54,83959	-1,87931	0,540475	1,82E-05	0,002013
Gpcpd1	2624,021	-1,92038	0,511584	6,41E-06	0,000873
Gm33543	488,3092	-1,92637	0,547484	1,55E-05	0,001792
2010003K11 Rik	1559,352	-1,92959	0,410739	1,09E-07	3,11E-05
Tbc1d30	195,2099	-1,94049	0,400325	5,23E-08	1,7E-05
Ackr2	83,12597	-2,04696	0,394511	9,33E-09	3,68E-06
BC030867	16,86352	-2,07126	0,621371	3,56E-05	0,003481
Nipal1	234,386	-2,10188	0,639891	3,37E-05	0,003345
Gm34667	178,3093	-2,15056	0,234967	2,9E-21	2,11E-17
Maff	147,1549	-2,24786	0,458449	3,87E-08	1,38E-05
3930402G23 Rik	126,417	-2,28048	0,465975	4,07E-08	1,41E-05
Zfp9	49,45074	-2,30592	0,381426	8,05E-11	5,59E-08
Apoa4	56978,27	-2,30783	0,44108	6,89E-09	3,05E-06
D930007J09 Rik	29,41297	-2,31383	0,454624	1,7E-08	6,42E-06
Adgrf1	23,14085	-2,42072	0,604582	3,04E-06	0,000486
Camk2b	286,5014	-2,4308	0,333998	1,6E-14	3,88E-11
Agap2	100,263	-2,70219	0,640322	9,29E-07	0,000184
9430037G07 Rik	96,81524	-2,82331	0,444986	1E-11	1,08E-08
Fabp5	475,0823	-2,86533	0,548276	7,47E-09	3,2E-06
Bcl2l14	32,03478	-3,02055	0,698622	7,75E-07	0,000161
Hspa1b	175,0326	-3,02118	0,638794	8,61E-08	2,56E-05
Gm45044	24,17073	-3,05581	0,761821	2,2E-06	0,000382
Trpm5	10,14297	-3,08019	0,775571	4,55E-06	0,000693

Cgref1	168,4962	-3,09017	0,510112	6,03E-11	4,62E-08
B930025P03 Rik	180,8033	-3,56638	0,415451	4,58E-19	2,23E-15
Cntnap1	275,9808	-3,73909	0,54044	2,05E-13	3,74E-10
2310034O05 Rik	19,39748	-4,20239	0,977043	3,32E-06	0,000527
Lepr	350,5264	-4,36665	0,521096	2,44E-18	8,88E-15
Ifi208	15,05129	-6,84088	2,590828	4,21E-07	9,44E-05
Gm43305	45,85173	-9,61292	2,946223	2,98E-11	2,72E-08

Supplementary table S4. Differentially expressed genes between WT and DUSP3-KO mice under HFD.

Gene name	Base Mean	log2 Fold Change	lfcSE	pvalue	padj
BC018473	61,0866786	10,91695	2,834897	1,85E-15	9,62E-12
Gm28438	10,46834075	6,566642	2,394893	5,8E-07	0,000377
Myo15b	105,3142347	4,697107	0,71147	1,89E-12	5,88E-09
Gm3776	49,59832018	4,486473	0,84043	4,24E-09	6,62E-06
Lrtm2	19,12604062	4,111087	1,193985	2,25E-05	0,00746
Cbr3	234,2000569	3,905198	1,109752	1,33E-05	0,005104
Serpina5	8,958231492	3,430901	0,849846	3,76E-06	0,001894
Fosb	50,29868366	3,41422	1,059139	3,86E-05	0,010859
Elovl7	42,50580592	3,330859	0,988412	2,45E-05	0,007812
Gstm3	2249,693528	3,173827	0,752752	9,56E-07	0,000597
Grip1	6,794487676	2,840346	0,887245	8,29E-05	0,018086
Fam83a	131,8984804	2,718401	0,432461	1,54E-11	3,43E-08
Folh1	13,1122516	2,585242	0,689632	7,96E-06	0,003548
Mogat2	17,11708882	2,547664	0,885596	0,000126	0,021689
Adgrv1	430,7786807	2,508338	0,521868	6,29E-08	4,67E-05
Sema3b	64,70072108	2,45375	0,358935	4,32E-13	1,68E-09
Bmp8b	10,52347643	2,313269	0,879665	0,000288	0,039826
Ankrd42	17,73108332	1,978109	0,548827	1,47E-05	0,005477
Slc39a4	430,0610957	1,937077	0,567166	2,21E-05	0,00746
Rragd	171,699426	1,911168	0,567367	2,66E-05	0,008154
Cidec	1961,579718	1,886692	0,337085	7,72E-10	1,34E-06

Mmp12	179,2855532	1,71705	0,584111	0,000108	0,02001
Sntg1	13,36001299	1,659308	0,654708	0,000383	0,048981
Ptch2	40,36462887	1,605692	0,524674	7,68E-05	0,017217
Srxn1	2149,932299	1,494234	0,564122	0,000243	0,035095
Ephb2	144,3724714	1,480255	0,593346	0,000372	0,048845
Mirt2	34,91713426	1,466158	0,544312	0,000223	0,033777
St6galnac2	49,53066354	1,438506	0,482104	9,86E-05	0,019487
Nqo1	393,9871405	1,429722	0,529037	0,000212	0,032776
Treh	101,5317918	1,411001	0,351654	2,44E-06	0,001271
Fndc10	23,51841293	1,407811	0,53685	0,000288	0,039826
Sh3pxd2b	71,72972036	1,377139	0,547466	0,00035	0,046748
Unc5b	185,618098	1,318643	0,416447	5,43E-05	0,013885
Casp12	143,0112333	1,300961	0,522697	0,000381	0,048981
Fgfr1	208,2057271	1,300426	0,525199	0,000383	0,048981
Tymp	860,3080675	1,296457	0,266077	4,75E-08	3,71E-05
Lad1	167,423654	1,24288	0,401378	6,84E-05	0,016942
Osbpl3	596,4851925	1,234203	0,383423	4,64E-05	0,012486
Mapt	222,5989308	1,223012	0,303906	2,41E-06	0,001271
Cyp2d12	28,90959319	1,180283	0,439383	0,000236	0,03486
Arhgef16	105,6210919	1,168274	0,231698	2,04E-08	1,99E-05
Gal3st1	129,5104305	1,125789	0,366952	7,72E-05	0,017217
Gstm2	1512,525438	1,123092	0,365843	7,66E-05	0,017217
Pgd	1143,995845	1,047009	0,39196	0,000245	0,035096
Usp20	306,5611425	1,015559	0,240859	1,06E-06	0,000638

Wdfy1	442,6565606	0,984349	0,191162	1,2E-08	1,57E-05
Ctps	118,7908149	0,980257	0,26375	8,36E-06	0,003548
Myh10	252,1580576	0,945156	0,275003	2,3E-05	0,007465
Nphp1	69,94903693	0,911811	0,336588	0,000233	0,03486
Gnai1	300,9708744	0,865652	0,285245	8,79E-05	0,018796
Tgfbr2	696,373036	0,865574	0,152244	6,49E-10	1,27E-06
Lama5	108,740818	0,843579	0,328636	0,000343	0,046428
Rcan1	472,8076574	0,797055	0,168434	9,96E-08	6,76E-05
Tsen54	176,5685237	0,676003	0,226886	0,000117	0,020727
Fam53b	236,0293108	0,664735	0,205909	5,21E-05	0,01378
Vat1	925,720313	0,644767	0,225974	0,00017	0,027212
Rab3d	353,8076959	0,638777	0,187846	2,93E-05	0,008634
Eno1	4597,515943	0,624823	0,150344	1,57E-06	0,000873
Gbe1	2579,163815	0,622656	0,217869	0,000171	0,027212
Acer2	341,9248383	0,619468	0,203208	9,48E-05	0,019356
Ank3	287,8938668	0,594505	0,19547	9,97E-05	0,019487
Nr6a1	203,337415	-0,62651	0,241399	0,000345	0,046428
Nlrp6	3006,42007	-0,65521	0,187239	1,89E-05	0,00659
Serpina1a	34149,6959	-0,65856	0,2546	0,000369	0,048845
Nhej1	100,5311091	-0,66069	0,210608	7,07E-05	0,016967
Ero1lb	965,5982263	-0,67008	0,223839	0,000107	0,02001
Tcp11l2	746,3992955	-0,70791	0,142682	3,33E-08	2,89E-05
Col5a3	1030,652624	-0,73691	0,292887	0,000386	0,048981
Zfp707	784,5892198	-0,75992	0,211995	1,34E-05	0,005104

Mir22hg	1456,472247	-0,78534	0,278729	0,000171	0,027212
Apom	4688,781263	-0,83062	0,214716	4,6E-06	0,002241
Hacl1	1241,431164	-0,91579	0,307814	0,000105	0,019944
Irgm2	1175,743814	-0,9383	0,34331	0,000206	0,032198
5830473C10Rik	892,7527128	-0,98148	0,323483	8,34E-05	0,018086
Smim1	143,0268519	-1,06118	0,354638	9,55E-05	0,019356
Dusp3	990,4969509	-1,10314	0,127462	2,44E-19	3,81E-15
1300014J16Rik	35,10537055	-1,12941	0,441115	0,000327	0,044803
Gm34667	178,3092712	-1,14874	0,242107	9,06E-08	6,42E-05
Mettl23	602,4434189	-1,17439	0,393544	9,46E-05	0,019356
Cadm4	148,0919277	-1,18315	0,349414	2,6E-05	0,0081
Sdf2l1	381,3050761	-1,22285	0,412523	9,99E-05	0,019487
Avpr1a	412,7772909	-1,29498	0,417731	6,53E-05	0,016445
Nfil3	714,5282473	-1,37755	0,268921	1,34E-08	1,61E-05
mt-Tm	157,8706374	-1,48941	0,46462	4,51E-05	0,012358
Hsd11b1	9931,882159	-1,48969	0,406618	8,41E-06	0,003548
Slc22a7	385,3787863	-1,5375	0,444584	1,9E-05	0,00659
Derl3	69,44341482	-1,58048	0,576	0,000181	0,028611
Gm12909	276,3702475	-1,60257	0,317735	1,9E-08	1,97E-05
Msmp	45,49975276	-1,95994	0,66764	0,000103	0,019853
Susd4	546,2535054	-2,09902	0,4049	8,93E-09	1,27E-05
Cyp2c37	7354,800764	-2,17959	0,531419	1,47E-06	0,000848
Gm48878	24,42694266	-2,24937	0,580645	4,74E-06	0,002241
Rdh16f2	2053,346624	-2,65817	0,349483	1,45E-15	9,62E-12

Snord13	28,53262405	-2,72045	0,551463	3,68E-08	3,03E-05
Ifi208	15,05129354	-3,58411	0,694562	1,79E-08	1,97E-05
Gm43305	45,85173154	-10,114	3,002079	3,15E-12	8,2E-09

Supplementary legends to figures

Figure S1. Concentrations of HDL (**a**) and LDL (**b**) the sera of DUSP3-KO and WT mice. Each dot represents one mouse. Mean ± SD values for all mice are shown. **P < 0.01; ***P< 0.001. (**c**) Representatives immunohistochemistry images illustrating the histological features that served for SAF scoring.

Figure S2. : (**a-b**) Representative images of livers from DEN-challenged DUSP3-KO and WT mice fed CD or HFD 24 weeks (**a**) and 32 weeks (**b**) post-DEN injection. (**c**) NAS score 24 and 32 weeks post-DEN injection. (**d-h**) Concentrations of T-CHO, LDL/HDL ratio and TG in the sera of mice 24 and 32 weeks post-DEN injection. Each dot represents one mouse. Mean ± SD values for all mice are shown. *P < 0.05; **P < 0.01; ***P< 0.001.

Figure S3. Concentrations AST (**a**) and ALT (**b**) in the sera of DEN challenged DUSP3-KO and WT mice fed CD or HFD after 24 and 32 weeks post-DEN injection. Each dot represents one mouse. Mean ± SD values for all mice are shown. *P > 0.05; **P < 0.01.

Figure S4. Heatmap representing differentially expressed genes between WT and DUSP3-KO mice under CD (a) and under HFD (b). Complete list of genes is in the supplemetary tables S3 and S4.

Figure S5. Expression of selected genes validated by qRT-PCR. RNA expression for each target gene was normalized to that of ribosomal protein S9 (RSP9). Results are expressed as mean ± SD. n=3 samples per condition.

Supplementary references

1. Dobin A, Davis CA, Schlesinger F, Drenkow J, Zaleski C, Jha S, et al. STAR: Ultrafast universal RNA-seq aligner. Bioinformatics. 2013;
2. Love MI, Huber W, Anders S. Moderated estimation of fold change and dispersion for RNA-seq data with DESeq2. Genome Biol. 2014;
3. Zhu A, Ibrahim JG, Love MI. Heavy-Tailed prior distributions for sequence count data: Removing the noise and preserving large differences. Bioinformatics. 2019;
4. Bahri MA, Plenevaux A, Warnock G, Luxen A, Seret A. NEMA NU4-2008 image quality performance report for the micropet focus 120 and for various transmission and reconstruction methods. J. Nucl. Med. 2009;
5. Bretin F, Warnock G, Bahri MA, Aerts J, Mestdagh N, Buchanan T, et al. Preclinical radiation dosimetry for the novel SV2A radiotracer [18F]UCB-H. EJNMMI Res. [Internet]. 2013;3:35. Available from: http://ejnmmires.springeropen.com/articles/10.1186/2191-219X-3-35

Figure S1

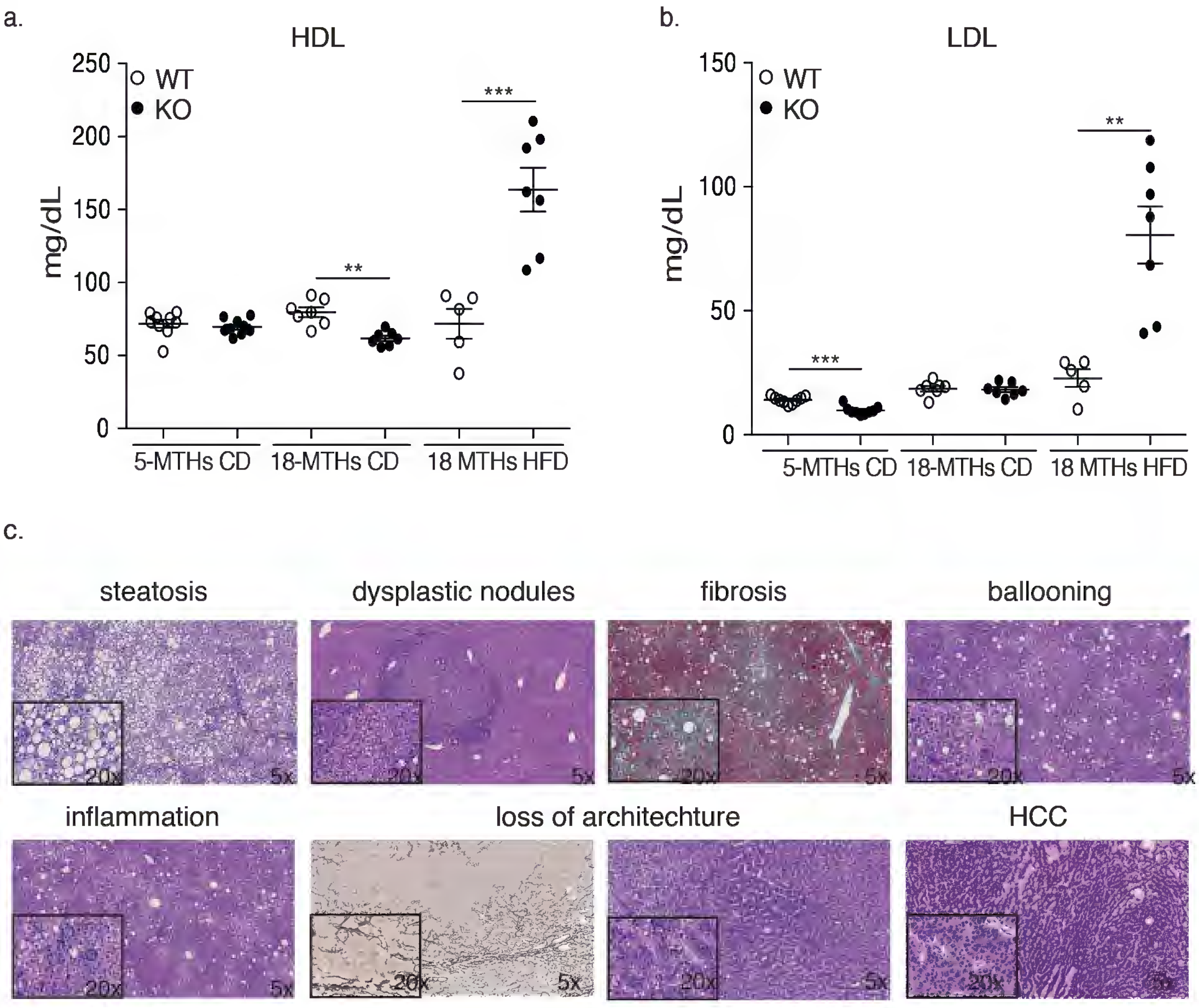

a.
HDL
250
200
150
100
50
0
mg/dL
WT
KO
***
**
5-MTHs CD
18-MTHs CD
18 MTHs HFD
b.
LDL
150
100
50
0
mg/dL
WT
KO
**
***
5-MTHs CD
18-MTHs CD
18 MTHs HFD
c.
steatosis
dysplastic nodules
fibrosis
ballooning
inflammation
loss of architechture
HCC
20x
5x

Figure S2

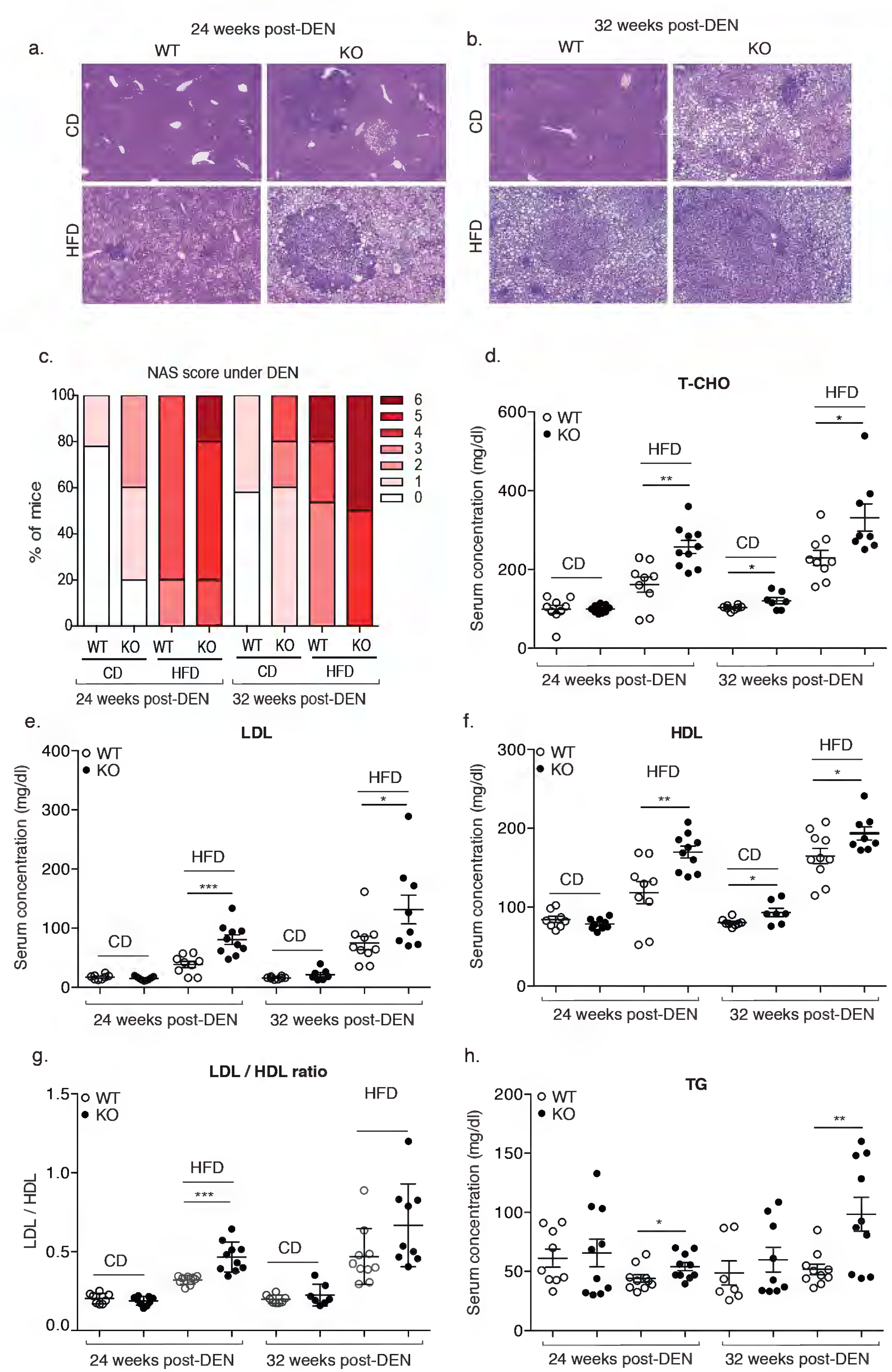

a.
24 weeks post-DEN
WT
KO
CD
HFD
b.
32 weeks post-DEN
WT
KO
CD
HFD
c.
NAS score under DEN
% of mice
100
80
60
40
20
0
6
5
4
3
2
1
0
WT KO WT KO WT KO WT KO
CD HFD CD HFD
24 weeks post-DEN 32 weeks post-DEN
d.
T-CHO
Serum concentration (mg/dl)
600
400
200
0
WT
KO
CD
HFD
**
*
24 weeks post-DEN
32 weeks post-DEN
e.
LDL
400
300
200
100
0
***
f.
HDL
300
200
100
0
g.
LDL / HDL ratio
LDL / HDL
1.5
1.0
0.5
0.0
h.
TG
200
150
100
50
0

Figure S3

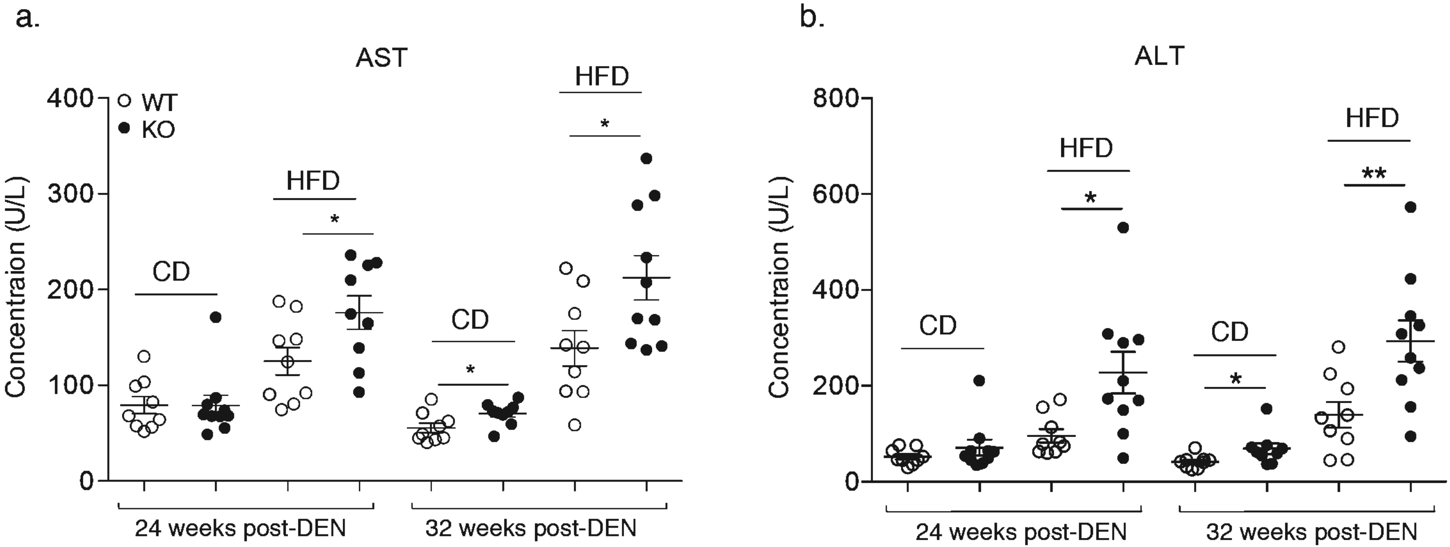
a.
AST
400
300
200
100
0
Concentraion (U/L)
WT
KO
CD
HFD
*
CD
*
HFD
*
24 weeks post-DEN
32 weeks post-DEN
b.
ALT
800
600
400
200
0
Concentraion (U/L)
CD
HFD
*
CD
*
HFD
**
24 weeks post-DEN
32 weeks post-DEN

Figure S4

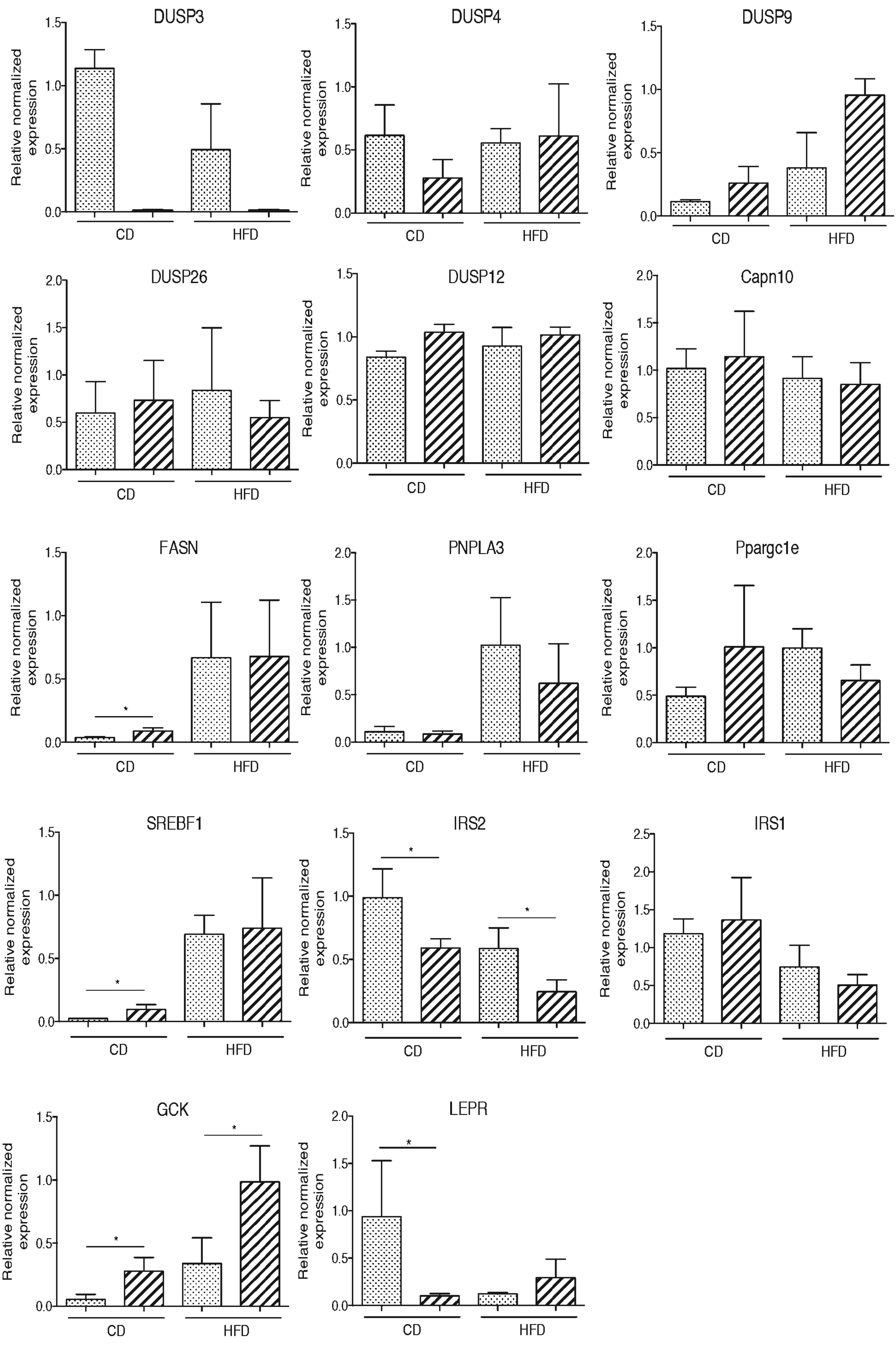
DUSP3
DUSP4
DUSP9
DUSP26
DUSP12
Capn10
FASN
PNPLA3
Ppargc1e
SREBF1
IRS2
IRS1
GCK
LEPR
Relative normalized expression
CD
HFD
*

Figure S5

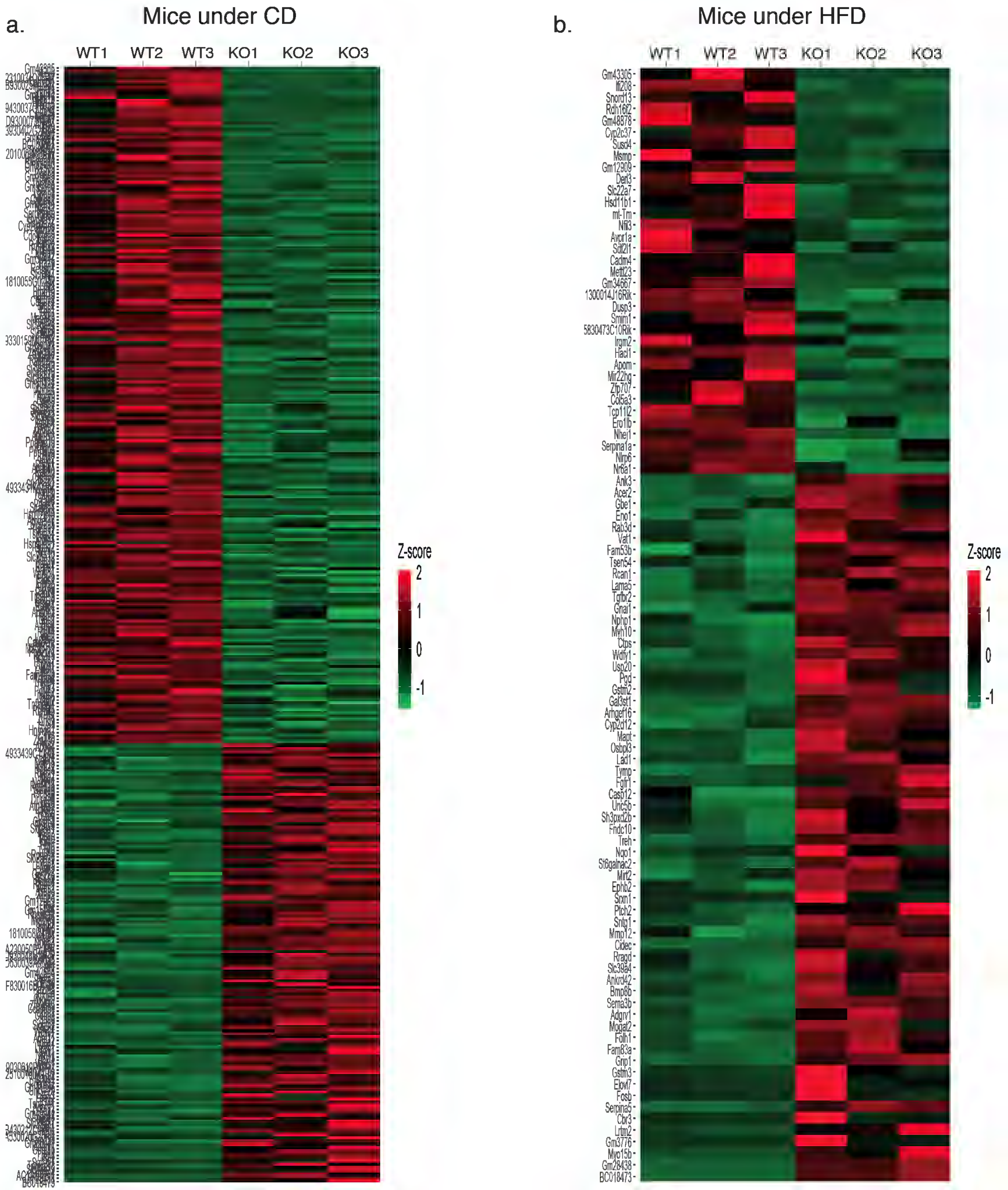
a.
Mice under CD
WT1
WT2
WT3
KO1
KO2
KO3
Z-score
2
1
0
-1
b.
Mice under HFD
WT1
WT2
WT3
KO1
KO2
KO3
Gm43305
Ifi208
Snord13
Rdh16f2
Gm48878
Cyp2c37
Susd4
Msmp
Gm12909
Derl3
Slc22a7
Hsd11b1
mt-Tm
Nfil3
Avpr1a
Sdf2l1
Cadm4
Mettl23
Gm34667
1300014J16Rik
Dusp3
Smim1
5830473C10Rik
Irgm2
Hacl1
Apom
Mir22hg
Zfp707
Col5a3
Tcp11l2
Ero1lb
Nhej1
Serpina1a
Nlrp6
Nr6a1
Ank3
Acer2
Gbe1
Eno1
Rab3d
Vat1
Fam53b
Tsen54
Rcan1
Lama5
Tgfbr2
Gnai1
Nphp1
Myh10
Ctps
Wdfy1
Usp20
Pgd
Gstm2
Gal3st1
Arhgef16
Cyp2d12
Mapt
Osbpl3
Lad1
Tymp
Fgfr1
Casp12
Unc5b
Sh3pxd2b
Fndc10
Treh
Nqo1
St6galnac2
Mirt2
Ephb2
Stxn1
Ptch2
Sntg1
Mmp12
Cidec
Rragd
Slc39a4
Ankrd42
Bmp8b
Sema3b
Adgrv1
Mogat2
Folh1
Fam83a
Grip1
Gstm3
Elovl7
Fosb
Serpina5
Cbr3
Lrtm2
Gm3776
Myo15b
Gm28438
BC018473
Z-score
2
1
0
-1
